# ARTGEL: A temperature-regulated gel electrophoresis platform for quantitative studies of reversible association

**DOI:** 10.1016/j.ohx.2026.e00847

**Published:** 2026-10-17

**Authors:** Rupam Saha, Seth Fraden

**Affiliations:** Martin A. Fisher School of Physics, Brandeis University, Waltham, MA 02453, USA

**Keywords:** EMSA, Gel electrophoresis, Chemical kinetics, Binding equilibria, DNA nanotechnology

## Abstract

Here we present ARTGEL, an actively regulated-temperature gel electrophoresis platform designed for quantitative studies of temperature-sensitive reversible biomolecular association. Existing literature describes electrophoretic mobility shift assays in which biomolecular reactions are suppressed during electrophoresis through dilution, the use of competitors, or reduced temperature. These approaches can alter temperature-sensitive equilibria and may cause the gel to report a state different from that of the original bulk solution. Rather than attempting to quench reactions, ARTGEL enables electrophoresis to be performed at the same temperature as complementary bulk measurements, allowing reversible association to be quantified directly in the gel and compared with matched measurements in solution. The ARTGEL platform integrates feedback-controlled thermoelectric regulation of gel temperature, a large, heated, and continuously circulated buffer reservoir to help maintain ionic strength, and automated electrode-wiping to mitigate electrode fouling and maintain stable electrophoresis current during long-duration electrophoresis. During electrophoresis, ARTGEL maintained the gel temperature at 30°C setpoint with a temporal variation of 0.03°C and a spatial range of 0.6°C. With periodic automated electrode wiping, the electrophoresis current remained at 139.5 ± 2.5 mA during the 24-h experiment. Using DNA origami assemblies, we further show that ARTGEL preserves distinct temperature-dependent association states, resolves reaction-dependent distortions of migrating bands, and supports extraction of in-gel kinetic and thermodynamic parameters from reaction–diffusion–advection modeling.

## Graphical abstract

**Figure:**
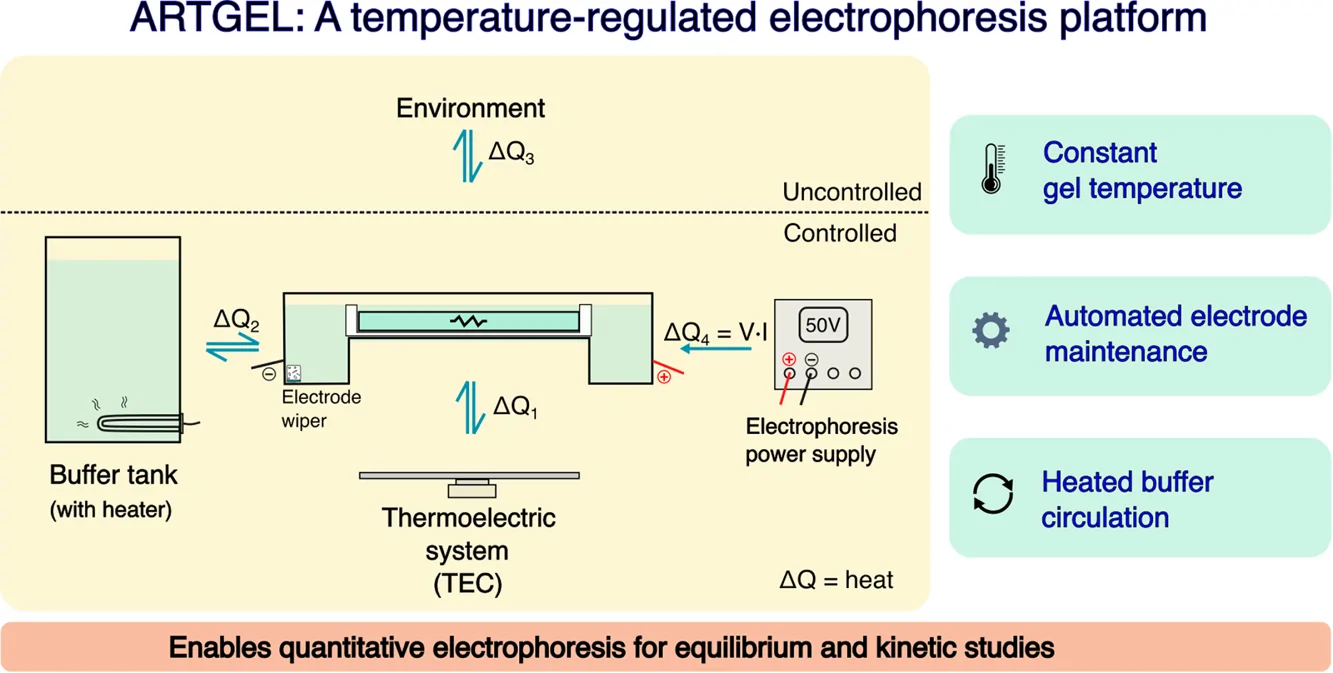

## Specifications table

**Table:** 

**Hardware name**	*ARTGEL*: An actively regulated temperature platform for agarose gel electrophoresis
**Subject area**	*Biological sciences*
**Hardware type**	*Biological sample handling and preparation*
**Closest commercial analog**	*Labsmith uProcess uEP01, Helena spife touch.*
**Open source license**	Hardware: CERN-OHL-P; Software: MIT License; Documentation/figures: CC BY 4.0.
**Cost of hardware**	*$2462*
**Source file repository**	https://doi.org/10.5281/zenodo.22909426

## 1 Hardware in context

Gel electrophoresis is one of the most widely used laboratory techniques for separating nucleic acids, protein–nucleic acid complexes, and nanoscale biomolecular assemblies based on electrophoretic mobility. Charged molecules migrate through a porous gel matrix under an applied electric field, with their migration depending on their interactions with the matrix. In most laboratory settings, it is used as a rapid analytical method to report molecular size distributions or sample purity under convenient ambient conditions. By contrast, an electrophoretic mobility shift assay (EMSA) is used to analyze interacting species, often with the goal of extracting binding equilibria and, in some cases, kinetic information for reversible biomolecular complexes [1], [2], [3], [4], [5], [6], [7], [8], [9], [10], [11], [12], [13].

In its conventional form, EMSA is usually intended to report the equilibrium state established in bulk solution before loading. The common strategy is therefore to make the gel as kinetically silent as possible: association is suppressed by dilution or the use of competitors [14], [15], [16], and dissociation is reduced by lowering temperature or by chemical stabilization [17], [18]. This strategy assumes that the population distribution prepared before loading remains effectively unchanged during sample loading, entry into the gel, and subsequent migration.

In practice, that assumption is often difficult to justify. Sample loading and entry into the gel can require 10–20 min (Fig. S5), which may be sufficient for substantial re-equilibration, especially when the gel is operated at a temperature different from the incubation condition. Moreover, lowering the concentration to suppress association will reduce the signal-to-noise ratio, while lowering the temperature can increase association or alter the equilibrium itself. More generally, even when electrophoresis is performed carefully, it may be unclear whether the observed band pattern reports the pre-equilibrated bulk solution or a state that has evolved further inside the porous gel matrix.

Here, we are motivated by a different scientific question. Rather than attempting to make the gel kinetically silent, we seek to make it a well-defined and controllable reaction environment whose behavior can be compared directly with that of the bulk solution. In this framework, the gel is not treated as an inert readout of a pre-equilibrated sample: confinement may alter $k_{\mathrm{on}}$, $k_{\mathrm{off}}$, and, in principle, $K_{d}$. ARTGEL therefore enables two complementary types of measurements. First, because electrophoresis spatially separates monomers, dimers, fragments, aggregates, and higher-order assemblies, it directly tests whether the assumed association model is adequate. Second, by operating at the same temperature as matched bulk measurements, ARTGEL allows the in-gel equilibrium and rate constants to be measured and compared with their solution values. Thus, unlike conventional EMSA, which primarily seeks to preserve the initial population distribution, ARTGEL uses electrophoretic separation to simultaneously resolve molecular species and quantify their evolution during transport.

DNA origami provides an excellent model system for demonstrating the importance of controlled electrophoretic conditions. Introduced by Rothemund [19], DNA origami uses a long scaffold strand folded by many shorter synthetic strands into prescribed nanoscale geometries. In the present work, we use DNA origami building blocks as a model system for studying reversible monomer–dimer association in an agarose gel environment. By measuring the in-gel equilibrium and rate constants, we investigate how confinement within the gel environment influences the equilibrium association state and the kinetics of the reaction.

To pursue this approach quantitatively, electrophoresis must be performed under conditions where temperature, electric field, and buffer composition remain well controlled over long durations. Under such conditions, the evolution of reactive species inside the gel can be analyzed by fitting spatial intensity profiles measured as a function of time to reaction–diffusion–advection models [7], [20], [21], [22], [23], [24]. This requires, at minimum, (i) a constant and spatially uniform temperature, because reaction rates are highly temperature sensitive, and (ii) a stable electric field, because the drift velocity enters directly into the transport model. For long experiments in high-salt buffers, maintaining approximately constant ionic conditions is also important for reproducible migration, thermal control and maintaining constant interparticle interaction strength.

Conventional electrophoresis platforms are not designed to meet these requirements. Joule heating in buffers containing added salt, including MgCl_2_-containing buffers used for DNA origami, raises the gel temperature, broadens bands, and perturbs both mobility and reaction kinetics [6], [25], [26], [27], [28]. Attempts to regulate temperature by adjusting electrophoresis power inherently couple thermal control to electric-field strength, preventing independent control of these variables [29]. In addition, prolonged electrophoresis in high-salt environments promotes electrode fouling through electrochemical precipitation, for example Mg(OH)_2_ in the presence of MgCl_2_, which increases electrode resistance, changes the effective voltage across the gel (SI section 1), and alters buffer composition over time. These effects compromise quantitative reproducibility during long-duration runs. Although temperature-controlled electrophoresis systems including Peltier-based regulation have been developed [30], these systems primarily address thermal control and rapid electrophoretic separation. They do not necessarily provide the combination of thermal regulation, electrode maintenance, long-duration electrophoresis, stabilization of electrophoresis current, and quantitative analysis of reactions occurring concurrently with electrophoretic transport. A detailed comparison of ARTGEL with the existing platforms can be found in Table 1 in the supplementary text.

To address these limitations, we have developed ARTGEL, an **a**ctively **r**egulated-**t**emperature **g**el **el**ectro-phoresis platform for long-duration experiments that is designed to provide independent control of gel temperature and applied electrophoresis conditions. Active temperature regulation provides a means of maintaining a desired thermal environment despite changes in heat generation or heat exchange with the surroundings, and is widely used in thermally controlled microfluidic and analytical systems [31]. In ARTGEL we implement a feedback-controlled temperature system and temperature sensors to continuously measure the system temperature and compare it with a desired setpoint. The difference between the measured and desired temperatures is used by a controller to adjust the heating or cooling power. ARTGEL integrates (i) direct thermoelectric regulation of gel temperature using a Peltier module coupled to an aluminum heat-spreading plate under proportional-integral (PI) control, (ii) an actively heated and continuously circulated large volume of buffer to reduce the thermal load on the Peltier module, improve spatial temperature uniformity, and minimize the decrease in ionic strength (SI section 2), and (iii) an automated electrode-wiping mechanism that mitigates salt deposition and stabilizes electrode resistance. Together, these features create a controlled in-gel environment suitable for extracting in-gel kinetic and thermodynamic parameters and for comparing them directly with complementary bulk measurements [32]. Thus, the distinguishing feature of ARTGEL is not temperature control alone, but the integration of thermal regulation, long-duration electrophoretic stability, and quantitative reaction analysis within a single electrophoresis platform.

ARTGEL is modular, fully documented and constructed from widely available components. All hardware designs, firmware, and control software are openly shared, enabling reproduction and customization. By transforming gel electrophoresis from a primarily qualitative separation method into a controlled platform for reactive transport in porous media, ARTGEL expands the utility of gel-based assays for quantitative studies of reversible biomolecular association, confinement effects, and temperature-sensitive nanoscale assemblies (see Fig. 1).

**Fig. 1:**
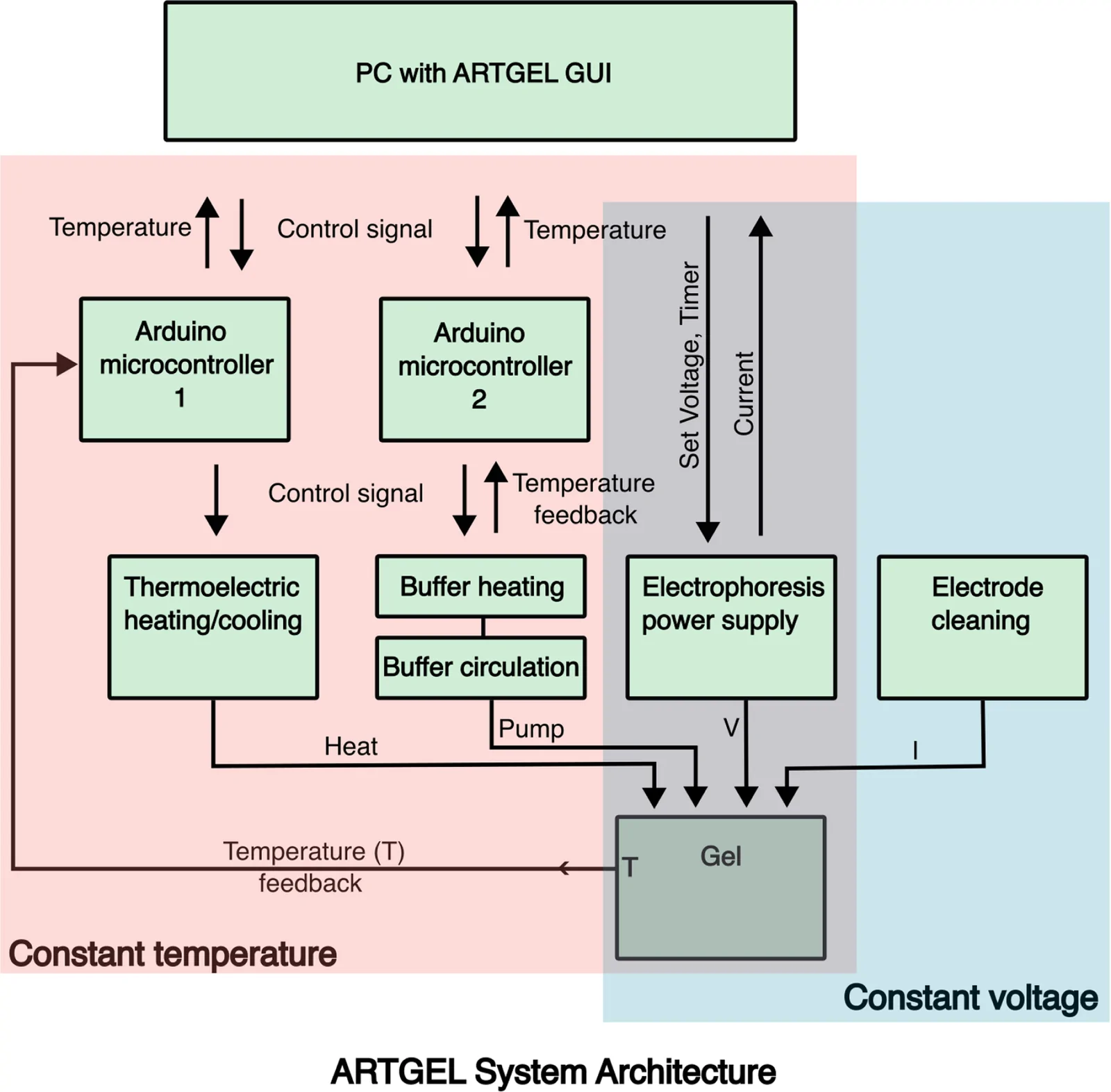
**Schematic of the gel temperature control architecture.** Red denotes parts of the system that contribute to controlling the gel temperature through the flow of heat. Blue denotes system elements that control the gel voltage. The gel receives four physical perturbations from the ARTGEL that affect its temperature; two are heat from the thermoelectric and circulating buffer, two set the electrophoretic electrical dissipation, VI, in which the electrophoresis power supply sets the voltage, V, while automated electrode wiping mitigates electrode fouling and helps maintain a stable electrophoresis current, I. Passive heat exchange with the environment is not indicated.

**Fig. 2:**
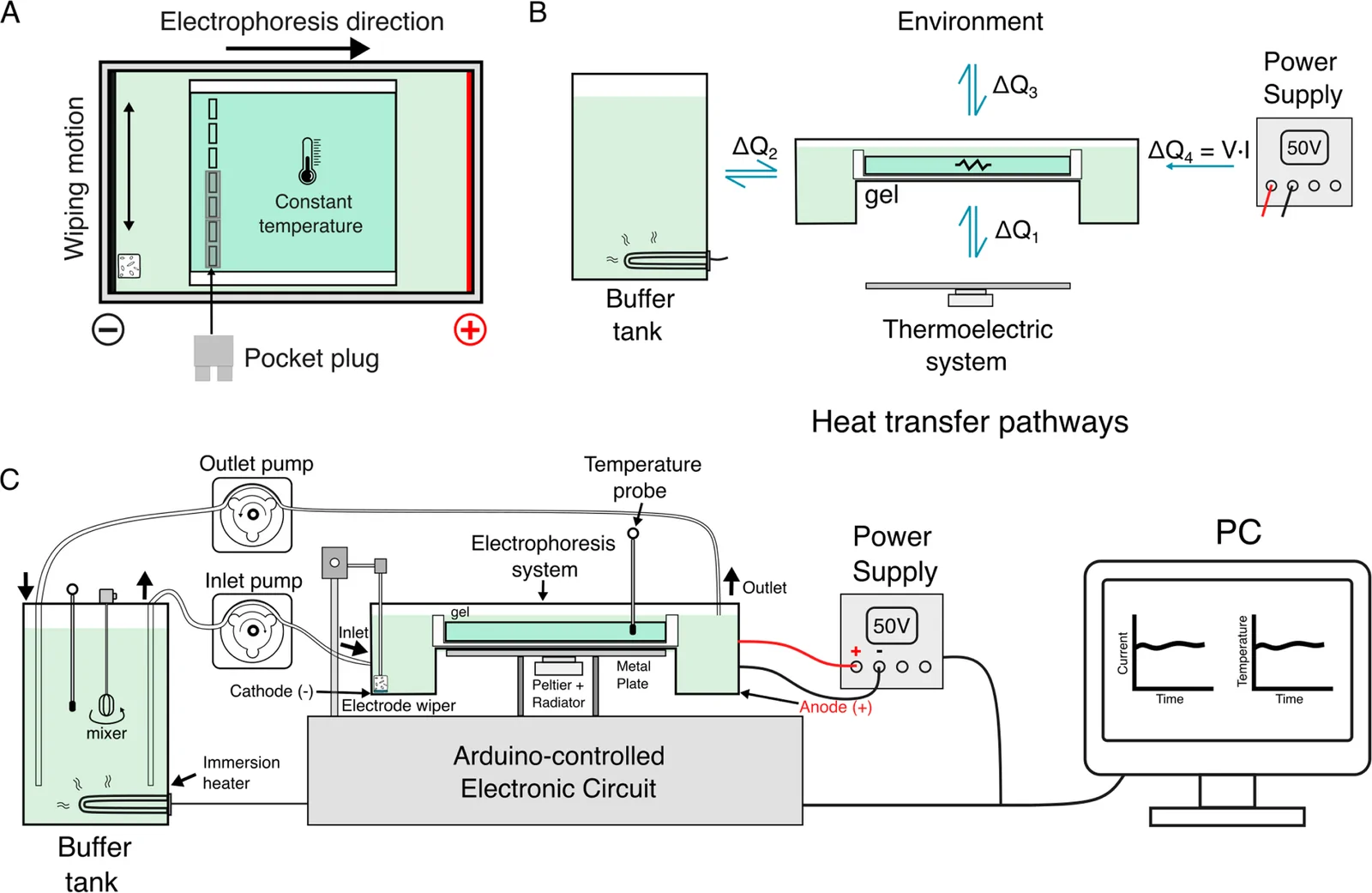
**System layout** A. Top-down view of the gel during electrophoresis under controlled temperature and voltage conditions. B. Schematic of heat-transfer pathways between the gel and surrounding components and electrical heat generation, including the buffer tank, thermoelectric system, external environment and electrophoresis power supply. C. ARTGEL schematic.

**Fig. 3:**
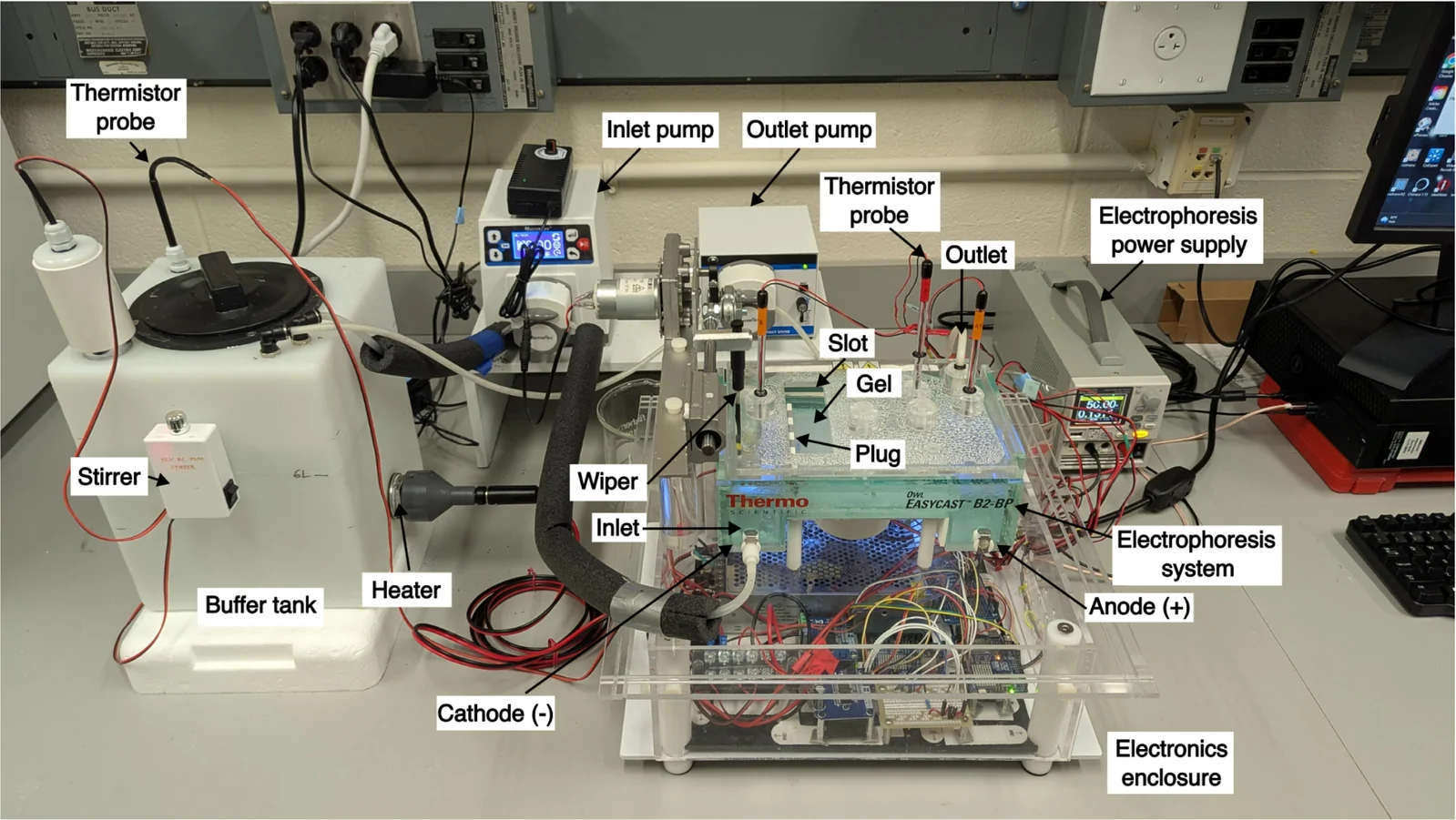
Instrument.

## 2 Hardware description

### 2.1 Hardware design and construction

In ARTGEL during electrophoresis we consider the gel to thermally interact with four components; the circulating buffer flowing over the gel, the thermoelectric (Peltier) module mounted outside and just below the gel, the electrophoresis power supply, which generates a voltage drop across the gel and concomitantly, resistive heating of the gel, and the ambient lab environment to which heat from the gel leaks (Fig. 2B). In our applications, the gel is typically operated about 10°C above room temperature. In this circumstance, in order of thermal mass, the gel is primarily heated by the circulating warm buffer and next the electrophoresis power supply. For active temperature control we employ a thermoelectric module under the gel which can actively supply or remove heat from the gel to regulate the gel temperature at the desired set point. Electrophoresis current is maintained by periodically removing salt deposits from the negative electrode by an automated wiping mechanism, described in more detail in Section 2.1.3. The operation of the complete ARTGEL instrument is shown in Supplementary Movie S1.

#### 2.1.1 Mechanical architecture

The electrophoresis part of the ARTGEL system is physically organized as a stacked modular assembly (Fig. 2C). The gel electrophoresis box is mounted on top of a water-resistant electronics enclosure (Fig. 3, Fig. 7B). The enclosure is constructed using Plexiglas sheets and nylon standoffs and houses all control electronics including microcontrollers, motor drivers (H-bridge), breadboards and power supply (Fig. 6). Ventilation openings on the enclosure walls allow airflow for thermal dissipation.

The top surface of the enclosure extends beyond the side walls and incorporates integrated drainage channels like moats on both the upper and lower faces (7B). These channels guide accidental liquid spills toward a corner reservoir, preventing fluid ingress into the electronics.

A flat aluminum heat-spreading plate is attached to the bottom of the gel electrophoresis box and is mounted on four standoffs on top of the electronics enclosure. A thermoelectric module and radiator assembly are attached to the underside of this plate. The area of the plate matches the area of the gel box to promote spatially uniform heat transfer (Fig. 2C).

#### 2.1.2 Thermal regulation subsystem

Thermal regulation in ARTGEL is implemented using a two-stage approach. Coarse temperature control is achieved by circulating temperature-controlled buffer from an external reservoir through the gel box. The buffer tank contains a 150 W immersion heater. Typically we set the buffer tank at a temperature, 0 – 2°C above the gel setpoint. Heat is also supplied by electrophoresis itself. We typically operate at 50 V, using 20 mM MgCl_2_ buffer, generating a current of about 140 mA, providing 7 W of heat to the gel. Fine temperature adjustment is provided by a 24 W thermoelectric (Peltier) module coupled to the aluminum plate beneath the gel, which is used to heat or cool the gel to the desired set point.

Prior approaches to temperature regulation in gel electrophoresis have relied on circulating heated buffer to regulate gel temperature [33], [34]. While effective in delivering heat, large buffer volumes introduce substantial thermal inertia, leading to slow response times and limited tunability. Conversely, thermoelectric control alone offers rapid adjustment but lacks the thermal uniformity provided by bulk buffer. In ARTGEL, these components work synergistically: the buffer reservoir provides thermal stability and spatial uniformity, while the thermoelectric module enables rapid and precise temperature tuning.

Temperature is monitored using multiple 100kΩ thermistors positioned at key locations, including within the gel, in the aluminum plate, in the buffer reservoir, and in the ambient environment. Each thermistor is entirely electrically insulated. Those embedded in the gel are housed in a cylindrical plastic enclosure with a nickel tip to provide good thermal contact with the gel, as well as corrosion resistance (Fig. 7A).

The thermoelectric module can be controlled manually, or automatically with proportional or proportional–integral feedback implemented on a microcontroller, referred to as Arduino 1. The buffer tank is controlled by a separate PI controller referred to as Arduino 2 (see SI section 3). A thermal safety switch rated at 65°C is attached to the thermoelectric heat spreading plate to prevent overheating from user or software errors.

#### 2.1.3 Electrophoresis and electrode maintenance subsystem

Electrophoresis is performed in a standard Thermo Scientific Owl Easycast B2-BP horizontal agarose DNA device. The device has two electrodes, each located in a deep buffer-filled well. The agarose gel, 1 cm thick, separates the two electrodes. One can model the effective resistance of the material between the electrodes in a lumped circuit model consisting of three resistances; the resistance between the anode and the gel, the resistance across the gel and the resistance between the gel and the cathode. We want the voltage drop across the gel to be constant so that the molecules migrate at constant velocity. However, the constant voltage power supply acts between the electrodes instead of across the gel. Consequently, if either of the electrode resistances changes with time, then the voltage drop across the gel will also change in time, even if the voltage between the electrodes remains constant. Because ARTGEL is an externally driven electrolytic cell, the cathode is the negative electrode and the anode is the positive electrode. Our buffer contains MgCl_2_, which leads to the electrochemical deposition of a thick, white coating of Mg(OH)_2_ on the cathode, which increases the electrode resistance, thereby lowering the current and, consequently the voltage drop across the gel. The ARTGEL system mitigates this effect by removing accumulated salt at the negative electrode using a custom-built reciprocating linear actuator (Fig. 3, Fig. 7F).

The actuator drives a scrubbing sponge attached to the wiper that periodically translates along the length of the negative electrode. The stroke length is adjustable, allowing the sponge to contact the full electrode length. The wiping frequency is set to approximately one stroke every five to ten seconds, which is found to effectively prevent salt buildup during long runs. The stroking speed is controlled using an integrated potentiometer.

We also designed and 3D printed plugs that fit into the wells of the gel. The purpose of the plugs is to prevent the scraped solid salt particles from the negative electrode from entering the gel. See SI section 5 for more details.

A commercial programmable electrophoresis power supply is used to operate the gel under constant voltage, and the voltage and the current are logged in real time.

#### 2.1.4 Buffer circulation and reservoir

Continuous buffer circulation is provided using an external buffer reservoir (capacity ≈7.6 L) connected to the gel box via tubing and two peristaltic pumps (Fig. 2C and 3). The total tube length is approximately 1 m and the tubing volume is approximately 30 mL. Buffer enters the gel box through an inlet port on the side wall and exits through outlet tubing inserted into an outlet port integrated into the lid (Fig. 2C). The height of the outlet tubing opening sets the height of the buffer in the gel box. The inlet pump is run at 30 mL/min. The outlet pump is operated at a higher flow rate than the inlet pump to maintain the buffer level within the gel box and prevent overflow. Inside the reservoir, a 150 W electrical immersion heater provides bulk heating, while a DC motor-driven stirrer promotes uniform buffer temperature (Fig. 7C). Cooling is passive through heat loss to the environment. The buffer temperature is measured using an independent thermistor and regulated using a dedicated microcontroller, referred to as Arduino 2.

#### 2.1.5 Electronics and control hardware

ARTGEL uses two Arduino-based microcontrollers operating independently, referred to as Arduino 1 and 2. Arduino 1 regulates gel temperature via the thermoelectric module (see TEC_control.ino in Section 3), while Arduino 2 regulates the buffer temperature in the reservoir (see Buffer_temp_control.ino in Section 3). Each Arduino interfaces with a motor driver (H-bridge) that switches the voltage polarity and, through PWM, sets the average voltage. The two H-bridges are powered by a shared 12 V, 250 W power supply, referred to as power supply 1. The Arduino uses pulse width modulation (PWM) to control the voltage to the thermoelectric. The PWM frequency is 976 Hz, which is much higher than the thermal response timescale of the gel box, so the temperature of the gel is set by the PWM value and not the instantaneous power.

Power supply 1 also drives the cooling fan and radiator assembly associated with the thermoelectric module. Five thermistors (A0^1^, A2^1^, A3^1^, A4^1^ and A5^1^) are connected to the gel temperature controller, while one thermistor is connected to the buffer temperature controller.

A computer interface enables real-time data logging and control via MATLAB. Custom software provides real-time visualization of temperature, voltage, and current data, as well as manual, closed-loop proportional and proportional-integral temperature control. See Fig. 4 for the pin configuration and system architecture.

**Fig. 4:**
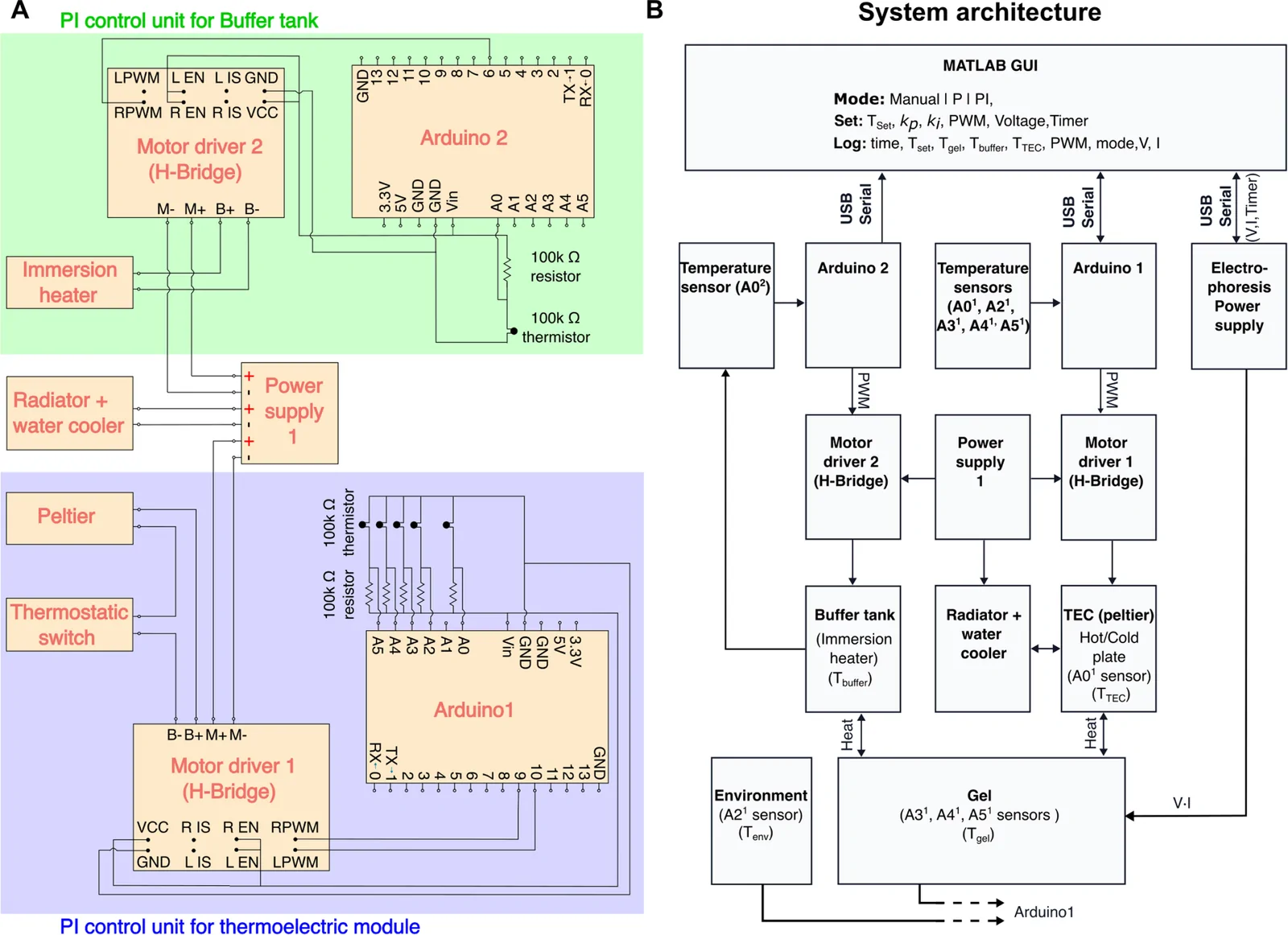
**Pin configuration and system architecture.** (A) Circuit diagram illustrating the Arduino pin configuration used to interface with the various electronic components. (B) Block diagram depicting the overall system architecture, showing how individual components interact with one another and are controlled via MATLAB.

### 2.2 Graphical user interface

The graphical user interface (GUI) is developed in MATLAB to provide centralized control, real-time monitoring, and automated data logging for the electrophoresis instrument (Fig. 5, see user_interface.mlapp supplied with design files (Section 3)). Through the GUI, users can specify key experimental parameters, including target gel temperature, control mode (manual, P, PI), electrophoresis voltage, and run duration. These inputs are transmitted to an Arduino-based controller via serial communication. During operation, the GUI continuously displays real-time measurements of electrophoresis current, gel temperature, and buffer temperature, together with the corresponding pulse-width modulation (PWM; 0–255) signals used for active temperature regulation.

Both open-loop and closed-loop temperature control strategies are implemented within the system. In manual control mode, the PWM value is directly set by the user, providing open-loop control of heating or cooling (Fig. 5A(i)). In PI control mode, the PWM output is automatically adjusted in a closed-loop to maintain the gel temperature at the desired setpoint (Fig. 5A(ii)). Live plots update dynamically throughout the experiment, enabling the user to assess temperature stability, transient responses, and controller performance in real time (Fig. 5B). All experimental parameters—including voltage, current, temperature, PWM, and time—are automatically recorded into structured data files for post-experiment analysis. In addition, integrated start and stop controls allow safe initiation and immediate termination of experiments. Together, these functions provide an interface that seamlessly integrates user input, hardware control, and quantitative data acquisition for temperature-regulated agarose gel electrophoresis.

**Fig. 5:**
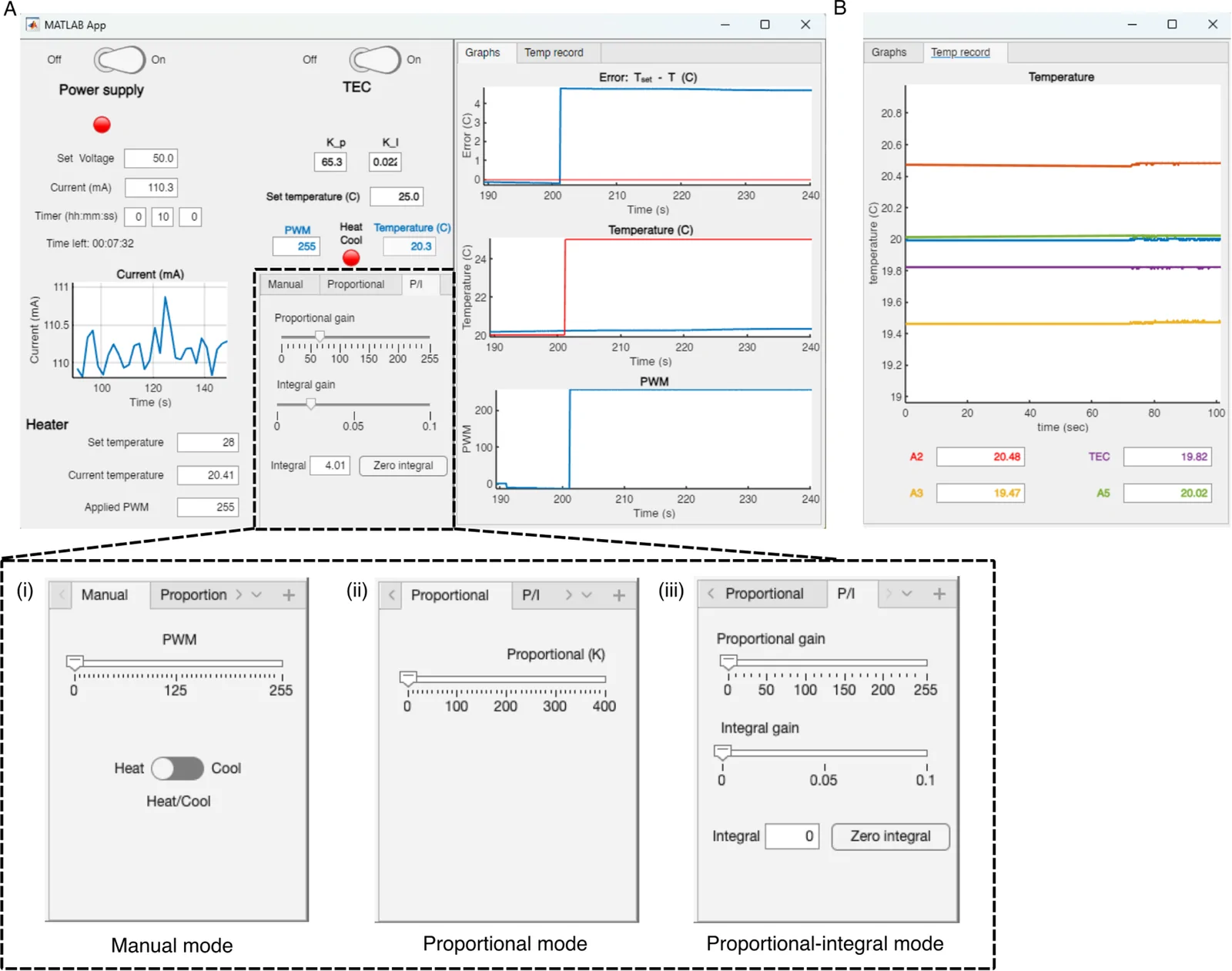
Graphical User Interface.

## 3 Design files summary

**Table:** 

**Design filename**	**File type**	**Open source license**	**Location of the file**

Buffer_temp_control.ino	Arduino code	MIT License	https://doi.org/10.5281/zenodo.22909426
TEC_control.ino	Arduino code	MIT License	https://doi.org/10.5281/zenodo.22909426
user_interface.mlapp	MATLAB GUI	MIT License	https://doi.org/10.5281/zenodo.22909426
plug.stl	STL file	CERN-OHL-P	https://doi.org/10.5281/zenodo.22909426

### 3.1 Buffer_temp_control.ino

This software file includes the Arduino code used to control and monitor buffer temperature by regulating the heater inside the buffer tank.

### 3.2 TEC_control.ino

This software file includes the Arduino code used to control and monitor gel temperature during agarose gel electrophoresis.

### 3.3 user_interface.mlapp

This software file includes the MATLAB graphical user interface used to control electrophoresis parameters, implement temperature feedback, and log experimental data in real time.

### 3.4 plug.stl

This file contains a 3D design of plugs to block the gel wells.

## 4 Bill of materials summary

The bill of materials is supplied in the form of a spreadsheet in BOM.xlsx and uploaded to the Zenodo Data Repository ( https://doi.org/10.5281/zenodo.22909426).

**Table:** 

**Component**	**Quantity**	**Cost per unit (USD)**	**Total cost (USD)**	**Source of materials**

Arduino Uno R4 WiFi	2	27.50	55.00	Amazon
BTS7960A H-bridge module	2	7.69	15.38	Amazon
Thermoelectric (Peltier) module	1	24.80	24.80	Digikey
Thermal switch	1	4.69	4.69	Digikey
100 kΩ NTC thermistor	6	2.45	14.70	DigiKey
100 kΩ resistor	6	0.10	0.60	DigiKey
Solderable breadboard	2	1.33	2.66	Amazon
Radiator + cooling fan	1	51.00	51.00	Amazon
Immersion heater	1	25.00	25.00	Amazon
DC power supply (12 V, 240 W)	1	30.38	30.38	Amazon
Power cord cable	1	15.39	15.39	Amazon
Terminal block	1	2.80	2.80	DigiKey
Nickel bar	1	13.19	13.19	Amazon
DC motor	1	6.89	6.89	Amazon
Scotch-Brite sponge	1	6.00	6.00	Amazon
Linear actuator	1	74.00	74.00	Amazon
Electrophoresis power supply	1	1107.00	1107.00	TEquipment
Peristaltic pump	2	69.00	138.00	Amazon
Pump tubing	1	10.00	10.00	Amazon
Buffer tank	1	53.98	53.98	U.S. Plastic Corp.
Gel electrophoresis system	1	811.00	811.00	Thermo Fisher Scientific

**Total**			**2462.46**	

**Fig. 6:**
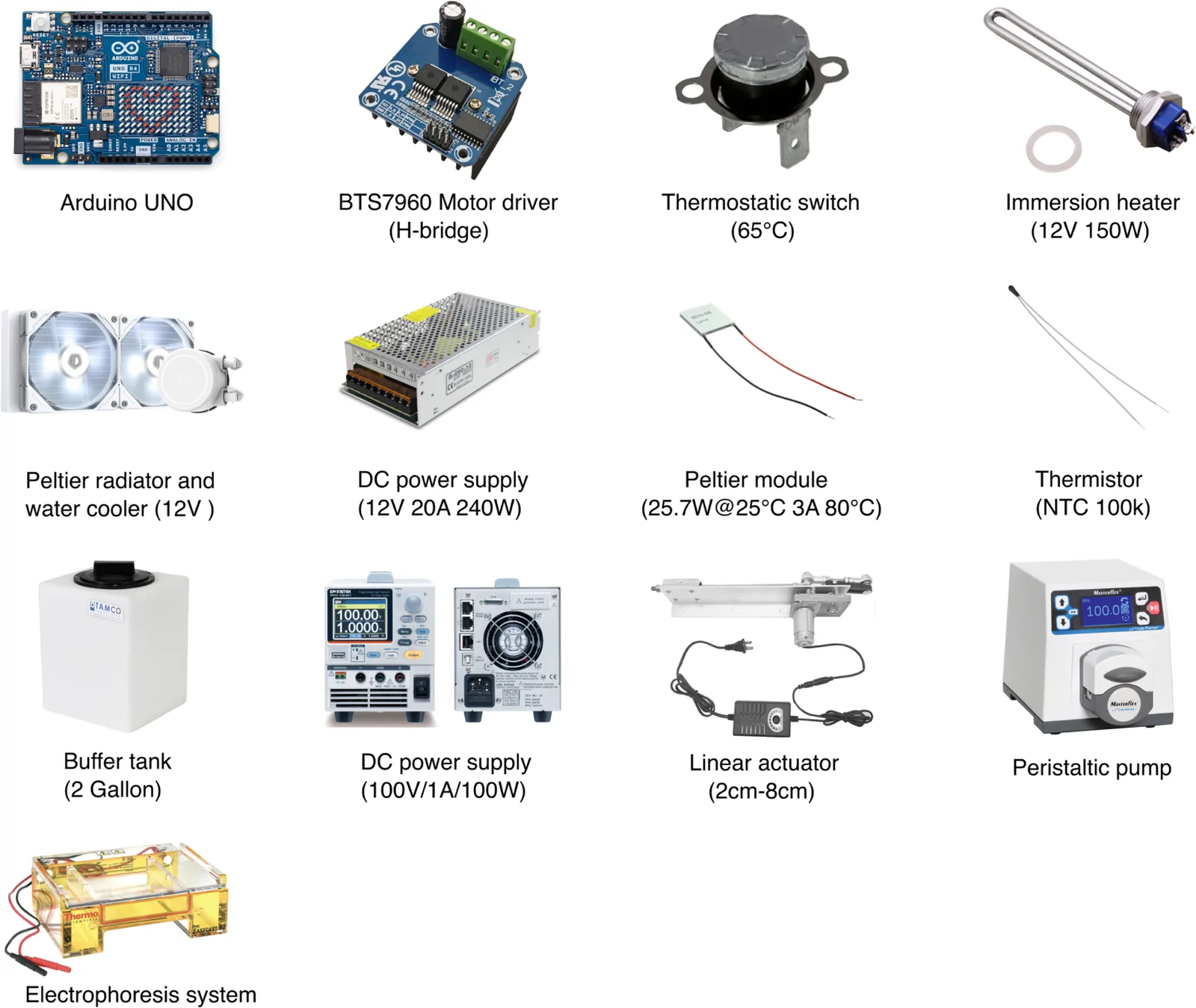
Commercial ARTGEL components.

## 5 Build instructions

1. To enable temperature monitoring during electrophoresis, six custom-fabricated thermistor probes are designed with appropriate electrical insulation and mechanical protection for operation in conductive and aqueous environments. The six probes are positioned to monitor the buffer reservoir (A0^2^), ambient room temperature (A2^1^), the aluminum plate (A0^1^), and three distinct locations within the gel for direct temperature measurement (A3^1^, A4^1^ and A5^1^). Of these, four probes (the three gel probes and the buffer reservoir probe) require electrical insulation due to direct contact with conductive or aqueous environments (Fig. 7A). Each thermistor is first soldered to 26-AWG insulated wires, and the solder joints are mechanically reinforced and electrically insulated using heat-shrink tubing. The wired thermistor is then inserted into a hollow plastic tube (approximately 6 mm in external diameter and 16 cm in length), with the thermistor leads exiting from the opposite end of the tube. A nickel tip, machined with an outer diameter slightly smaller than the inner diameter of the plastic tube, is used to house the exposed thermistor bead. Thermal adhesive is applied around the thermistor bead, which is then sealed inside the nickel tip to ensure good thermal contact while maintaining electrical isolation. The nickel tip is subsequently inserted or snap-fitted into the plastic tube. The open end of the tube is sealed using a rubber cap, which both secures the wiring and reduces mechanical stress on the leads. For the buffer reservoir probe, the plastic tube length was increased as required to accommodate the reservoir geometry and immersion depth.

Precaution: Make sure the nickel tip is tightly attached and sealed to the tube to prevent water leakage. One can use vacuum grease as sealant.

2. The electrical circuit inside the electronics enclosure consists of two Arduino microcontrollers, two motor driver (H-bridge) modules, two breadboards, six 100kΩ resistors, six 100kΩ thermistors, one terminal block, and a DC power supply (referred to as “Power supply 1”). The motor driver functions as a switch to reverse the direction of current, enabling the thermoelectric module to both heat and cool the gel. One Arduino–motor driver (H-bridge) pair is dedicated to controlling the thermoelectric (Peltier) module for gel temperature regulation, while the second Arduino–motor driver pair independently controls the immersion heater used for buffer temperature regulation. Of the six 100 kΩ resistors and six 100 kΩ thermistors, five resistor–thermistor pairs are connected to Arduino 1, while one pair is connected to Arduino 2. Each known resistor forms a voltage divider with its thermistor, allowing the Arduino to determine temperature from the measured voltage. See SI section 6 for a detailed description on thermistor calibration. Two solderable breadboards are used to keep the two control circuits electrically separated. All the components are assembled following the wiring schematic shown in Fig. 4A, with all control and power connections verified prior to integration. Power supply 1 is connected to a 10 A, 125 V power cord cable and provides power to the cooling fan–radiator assembly, the Peltier module via its motor driver, and the immersion heater via its corresponding motor driver. A terminal block is used to organize signal wiring, reducing clutter and simplifying troubleshooting. All electronic components are mounted on a rectangular plastic platform using standoffs and secured with hook-and-loop fasteners to ensure mechanical stability while allowing easy access for maintenance and modification.

3. The electronics enclosure is constructed using a stacked Plexiglas architecture supported by four machined nylon standoff pillars, each approximately 12 cm in height (Fig. 7B). The bottom rectangular base plate has dimensions of 40 cm × 30 cm, while the top plate is intentionally fabricated with a larger footprint and curved edges incorporating integrated drainage channels. These channels guide accidental liquid spills away from the electronics and toward an external collection reservoir. Each standoff pillar contains curved internal slots that allow the electronics mounting platform to slide into the enclosure during assembly. The left, right, and front enclosure walls are fabricated from perforated Plexiglas sheet to promote airflow, whereas the rear wall houses the cooling fans. The top plate is secured to the four main standoffs using screws. Four additional short standoffs (approximately 4 cm in height) are then mounted on the top surface of the enclosure to support the aluminum thermal plate. The aluminum plate is secured to these standoffs such that it sits above the enclosure roof, forming the thermal interface for the gel box. The thermoelectric (Peltier) module, thermal safety switch, and radiator are mounted to the underside of the aluminum plate. A hole is drilled into the aluminum plate from the edge to the center to house the thermistor (A0^1^) used to monitor the plate temperature.

**Fig. 7:**
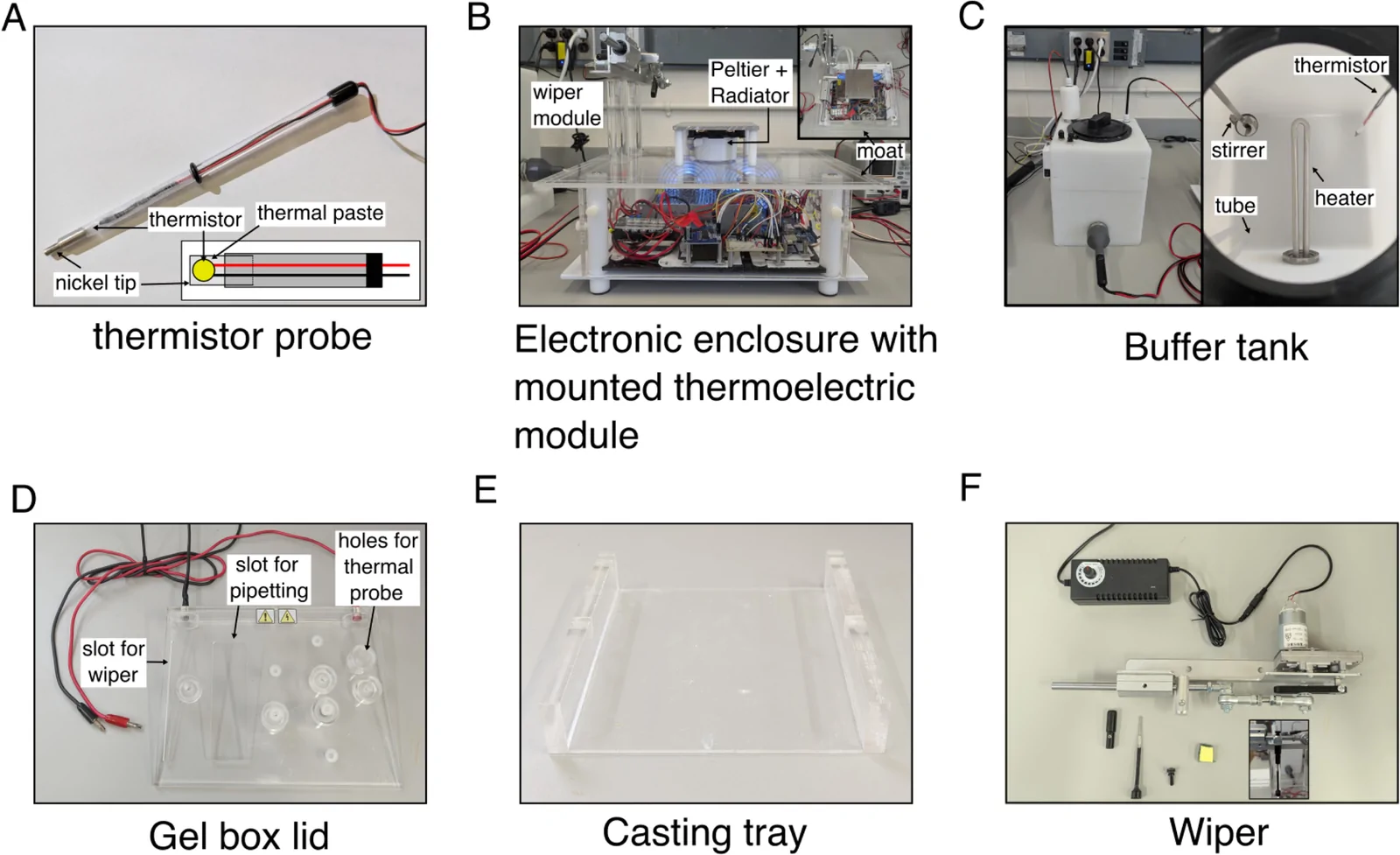
Customized ARTGEL components.

4. A 2-gallon (approximately 7.6 L) tank is used as the buffer reservoir (Fig. 7C). The immersion heater is mounted on one side wall of the tank approximately 3 cm above the bottom surface using a 1-inch NPSM (National Pipe Straight Mechanical) threaded flange. The heater has a female threaded interface. A custom male plug connected to a 14-AWG insulated wire is fabricated to provide power while maintaining a secure and serviceable electrical connection. On the top surface of the reservoir, four access ports are drilled: one port accommodates the thermistor (A0^2^) for measuring the buffer temperature, one is used to mount a 12 V DC mechanical stirrer for continuous mixing, and two additional ports serve as the inlet and outlet for buffer circulation tubing connected to the gel box. This configuration ensures uniform buffer temperature, reliable circulation, and ease of maintenance during long-duration electrophoresis experiments.

5. The gel-box lid and gel tray are modified to accommodate temperature probes, the electrode wiper, and efficient thermal coupling between the gel and the aluminum thermal plate (Fig. 7D and E). First, multiple holes are drilled into the gel box lid directly above the gel footprint and above the two buffer reservoirs, allowing thermistors to be inserted vertically into the gel and buffer from above without disturbing the electrophoresis setup. Near the negative electrode, a rectangular slot approximately 40 mm wide and 115 mm long is cut into the lid to allow passage of the stick holding the wiper, enabling continuous cleaning along the full length of the electrode during operation. The gel tray is then machined to reduce its thickness to approximately 1 mm, minimizing thermal resistance and improving heat transfer from the underlying metal plate to the gel. Finally, all air gaps are sealed using compressible foam boards to provide thermal insulation and maintain stable electrophoresis conditions during extended runs.

6. The reciprocating linear actuator is customized to function as an automated electrode wiper (Fig. 7F). Two rigid standoffs are fabricated and mounted on the top surface of the electronics enclosure adjacent to the negative electrode side of the gel box. The linear actuator is securely screwed to these standoffs to ensure stable and repeatable motion during operation. A stainless-steel rod (length 10 cm) with a thicker round base is fabricated such that one end threads directly into the actuator arm, while the opposite end extends through the previously machined slot in the gel box lid (Fig. 7D). At the bottom of this rod, we tape a velcro hook that applies gentle pressure to a sponge element (Scotch-Brite block of dimension 15 mm × 15 mm), transmitting the actuator’s motion. The rough surface of the sponge is oriented toward the negative electrode, where it gently removes salt deposits that accumulate during electrophoresis. The stainless-steel rod is covered with heat-shrink tubing to protect against corrosion from the electrolyte and to provide electrical insulation. The actuator is powered at 24 V and includes an integrated potentiometer, which allows adjustment of the motor speed and consequently the wiping stroke frequency.

7. Two peristaltic pumps are installed to establish continuous buffer circulation between the external reservoir and the gel box (Fig. 2C). The inlet line from the buffer tank is routed through one peristaltic pump and delivers heated buffer into the gel box through a side port. A second line (outlet) from the gel box is routed through another peristaltic pump and the buffer is delivered back to the buffer tank. The outlet line is designed to exit the gel box vertically through the lid. The height of the opening in the outlet tube sets the height of the buffer to be 1–2 mm above the surface of the gel (Fig. 2C). The inlet flow rate (30 mL/min) is regulated to provide steady buffer delivery, while the outlet pump is intentionally operated at a higher flow rate to maintain a constant buffer height within the gel box. To further suppress fluctuations in liquid level, the outlet tubing is coupled to a 10 μ L pipette tip, which acts as a flow restrictor. The inlet tubing is wrapped with insulating foam to minimize thermal losses during the transfer of heated buffer from the reservoir to the gel box.

8. Finally, the microcontrollers and the electrophoresis power supply are linked to the computer using USB cables to actively monitor and regulate the temperature, heating, voltage and current. A list of all the components, their prices and supplier information can be found in Section 4 or in BOM.xlsx.

## 6 Operation instructions

### 6.1 Buffer preparation and thermal control initialization

• Prepare the electrophoresis buffer according to the planned duration of the experiment.
• Fill the buffer tank and insert the thermistor probe into the reservoir.
• Connect the heater to the power supply.
• Turn on power supply 1 inside the electronics enclosure.
• Connect Arduino 2 to the desktop computer.
• Open Buffer_temp_control.ino program and:
  – Set the target temperature
  – Define the PI parameters (Kp, Ki)
  – Compile and upload the code
• Turn on the mechanical mixer to ensure uniform buffer temperature.
• Open the serial monitor and verify that the buffer temperature reaches and stabilizes at the target value.

### 6.2 Gel preparation and thermal coupling

• Prepare the gel following the standard protocol and allow it to fully set.
• Once the buffer temperature has reached and stabilized at the set value:
  – Place the gel on the machined casting tray
  – Position the gel box on the metal thermal plate
• Apply a thin layer of thermal paste between the gel box and metal plate to improve thermal contact.

### 6.3 Wiper system installation and verification

• Place the Scotch-Brite sponge on the negative electrode.
• Insert the stainless steel rod through the designated lid slot and press it gently against the sponge.
• Secure the rod to the arm of the reciprocating actuator.
• Turn on the actuator at minimum speed and verify that:
  – The stroke spans the entire electrode length
  – The electrode surface is effectively wiped
• Confirm that:
  – The actuator is mechanically stable
  – Alignment screws do not wobble
  – The wiping action is gentle and does not damage the electrode

### 6.4 System insulation and sensor placement

• Insulate the gel box using compressible foams, ensuring all air gaps are filled (Fig. S8).
• Insert thermistors into:
  – The gel
  – The metal plate
• Insert the thermistors into the gel to a depth of approximately three-quarters of the gel thickness (approximately 7.5 mm for a 1 cm-thick gel), measured from the top surface of the gel.
• Verify that all temperature sensors provide stable readings.

### 6.5 Buffer circulation setup

• Connect the buffer tank to the gel box using tubing.
• Turn on the peristaltic pumps to fill the gel box until the gel is fully submerged.
• Once the desired buffer height is reached:
  – Reduce the inlet pump flow rate to the target value
  – Set the outlet pump flow rate higher than the inlet flow rate
• Confirm that the buffer level in the gel box remains constant during circulation.

### 6.6 Electrophoresis initialization and sample loading

• Block the gel wells with the 3D-printed plugs (see plug.stl from Section 3).
• Connect Arduino 1 and the electrophoresis power supply to the computer.
• Open the GUI and make sure the serial communication is established.
• Set the voltage and timer.
• Start electrophoresis and begin monitoring gel temperature.
• Turn on the wiper.
• Allow the gel temperature to quickly rise. When the gel temperature is within 2°C of the target temperature, activate the PI temperature controller in the software.
• Once the gel temperature stabilizes at the target:
  – Turn off the electrophoresis power supply
  – Remove the desired plug
  – Pipette samples into the gel wells
  – Put the plug back into the well.
• Reset the experiment timer and restart electrophoresis to begin the run.

### 6.7 Run monitoring and shutdown

1. Monitor

• Gel temperature
• Buffer level
• Wiper operation

2. At the completion of the run:

• Turn off the electrophoresis power supply
• Stop the wiper system
• Turn off pumps, heaters, and mixers

## 7 Validation and characterization

The performance of ARTGEL was validated by assessing its ability to maintain stable gel temperature, minimize spatial temperature gradients, and maintain stable electrophoretic conditions during 24 h agarose gel electrophoresis of interacting DNA origami samples.

### 7.1 Temperature stability during agarose gel electrophoresis

We first examined the impact of unregulated gel temperature on temperature-sensitive DNA origami samples during agarose gel electrophoresis [35], [36], [37], [38]. The origami samples consisted of two controls, a “permanent” monomer without any designed interparticle bonds (size 50 nm) and a “permanent” dimer consisting of a pair of strongly bound monomers (Fig. 8A), and a reactive sample (Fig. 8B) in which the origami was capable of reversible monomer–dimer interconversion. The reactive samples were pre-incubated at temperatures ranging from 40°C to 30°C, allowed to establish distinct equilibrium states, and subsequently loaded into different lanes of the same gel at room temperature (23°C) without actively regulated gel temperature. Despite their different initial conditions, the electrophoresis results showed that all reactive samples converged to nearly identical intensity profiles, which, as shown in Fig. 8C, consists primarily of dimers. Note that after loading, DNA origami samples required approximately 10–20 min to enter the gel from the well, providing sufficient time for re-equilibration (Fig. S5).

When electrophoresis was conducted using ARTGEL under actively regulated constant gel temperatures, the initial thermodynamic states were preserved and directly resolved. DNA origami samples were incubated at temperatures ranging from 26°C to 32°C prior to electrophoresis. The reactive samples exhibit a monotonic increase in the dimer population with decreasing temperature (Fig. 8E–F), in contrast to the case when samples were run at 23°C and the distributions were the same, independent of incubation temperature, demonstrating that ARTGEL maintains a stable thermal environment which preserves the thermodynamic states during electrophoresis.

**Fig. 8:**
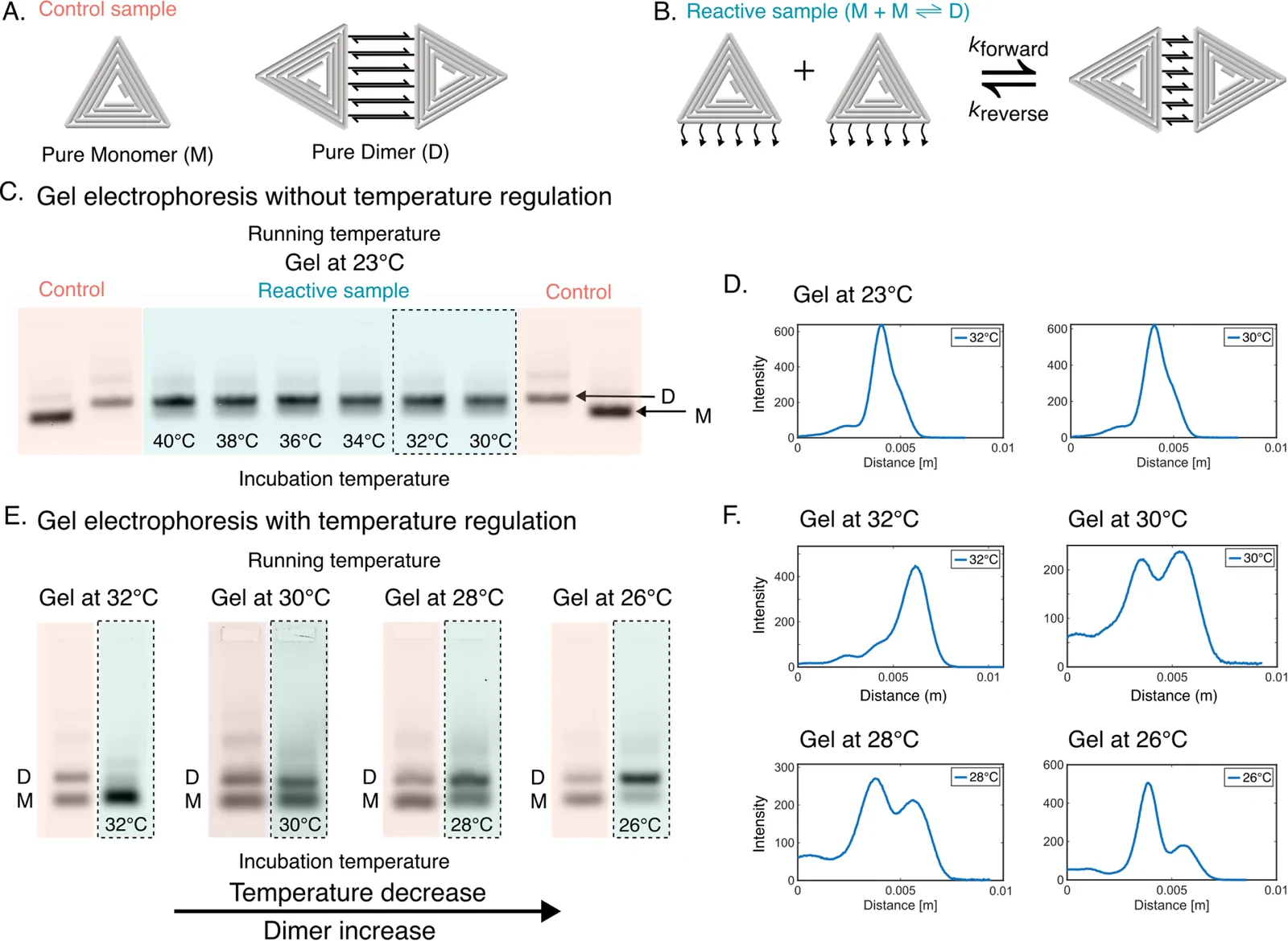
**Temperature dependent behavior of DNA origami building blocks in agarose gel electrophoresis.** A. Non-reactive DNA origami monomer and dimer as control samples. B. Reactive samples that include one side active monomer and dimer with a six-base-pairs-long hybridized domain for each overhang pair. C. Agarose gel electrophoresis at ambient temperature (23°C), without active temperature regulation. The orange and teal colored lanes contain the control and reactive samples respectively. Reactive samples were pre-incubated at different temperatures ranging from 40°C to 30°C. D. Intensity profiles of reactive samples incubated at 30°C and 32°C and run at 23°C. The corresponding lanes in panel C are indicated with a dotted rectangular box. E. Agarose gel electrophoresis conducted at actively regulated constant temperatures of 32°C, 30°C, 28°C and 26°C (from right to left). F. Intensity profiles of the reactive samples shown in panel E.

### 7.2 Temperature regulation during electrophoresis

Next, we evaluated the temperature-control performance of ARTGEL, conducting a 16-hour gel electrophoresis experiment at 50 V, with 1.5% agarose gel. The gel and buffer reservoir were regulated at set temperatures of 30°C and 31°C, respectively. The buffer temperature was maintained 1°C higher than the gel to compensate for heat loss during buffer circulation. We performed this experiment with a total of 7.2 L circulating buffer and the external buffer was not replaced during the experiment.

Both gel and buffer temperatures remained stable throughout the experiment. After excluding the initial 2 h required for equilibration (for gel), the 2-min-averaged gel temperature was measured to be 30.00±0.03°C (Fig. 9A), while the corresponding 2-min-averaged buffer temperature was measured to be 31.0±0.2°C (Fig. 9B). The reported standard deviations quantify the temporal variation of the measured temperature during the experiment and do not represent absolute temperature accuracy, calibration uncertainty, or spatial temperature uniformity. A more detailed description on the temperature-data acquisition and processing can be found in SI section 4.

The proportional–integral (PI) control parameters were set to $k_{p}=150$ and $k_{i}=0.03$ for the thermoelectric module associated with the gel, and $k_{p}=20$ and $k_{i}=5$ for the buffer module. The buffer PWM output exhibited slow oscillations with a period of approximately one hour, arising from non-optimized controller gains (Fig. 9B). However, due to the large thermal time constant of the buffer reservoir, these oscillations did not affect the buffer tank or gel temperature stability and could be further minimized through controller tuning if required. The PI parameters used for the thermoelectric gel-temperature controller were also not optimized for the experiment shown here; optimized parameters identified subsequently are provided in SI Section 3.

These results demonstrate that ARTGEL provides robust and stable temperature regulation during long-duration electrophoresis experiments.

**Fig. 9:**
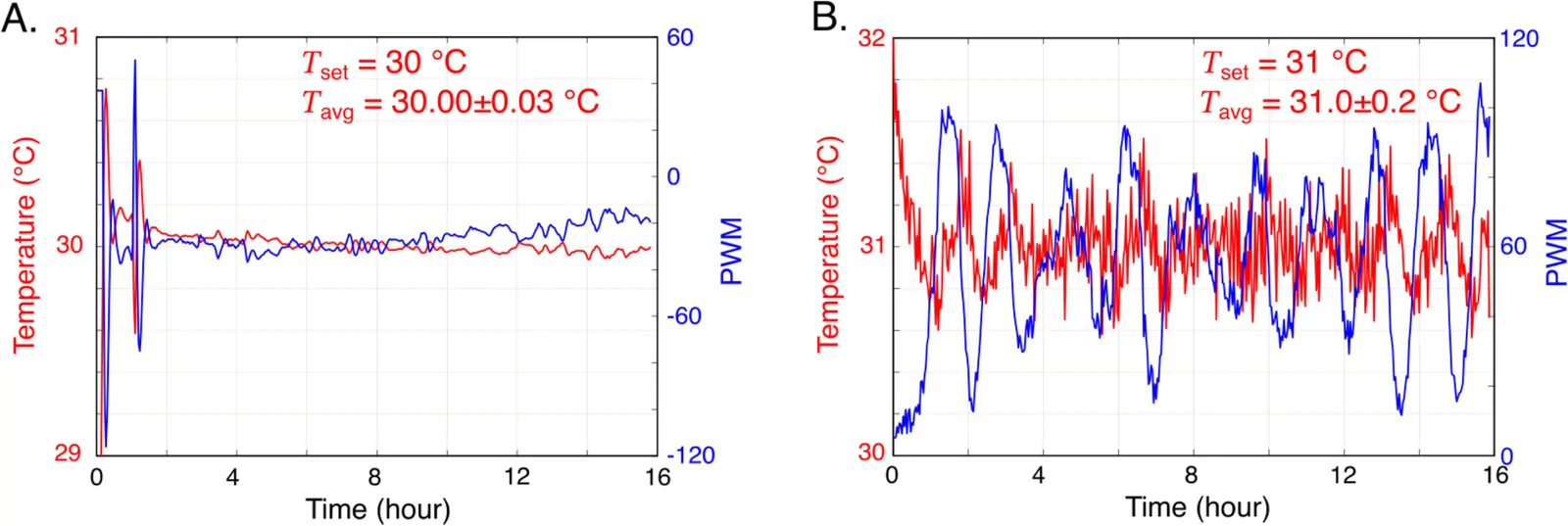
**Temperature regulation during agarose gel electrophoresis.** A. Temperature regulation of the agarose gel at a set temperature of 30°C. B. Temperature regulation of the buffer at a set temperature of 31°C. Temperature (red) and corresponding PWM control signal (blue) are plotted as a function of time. Both the temperature and PWM data are averaged over 2-min intervals for presentation. The reported $T_{\text{avg}}$ values represent the mean ± standard deviation of the 2-min-averaged temperature measurements. The mean and standard deviation of the gel temperature are reported after excluding the initial 2 h equilibration period.

### 7.3 Spatial temperature uniformity within the gel

Temperature uniformity across the gel is critical for preventing unintended spatial variation in electrophoretic mobility and reaction equilibria. First, we calibrated all the thermistors in the temperature range from 20°C to 44°C. The calibration procedure is described in the supplementary section 6. We then measured gel temperature simultaneously at three locations during electrophoresis under active regulation at setpoints of 30°C and 40°C (Fig. 10A), with the T3 sensor serving as the feedback-control sensor. In both cases, the buffer temperature was maintained slightly above the gel temperature, which helped compensate for the heat loss during buffer circulation. For precise locations of the thermistors a dimensioned diagram is provided in supplementary (SI Fig. S9).

At a 30°C setpoint (Fig. 10B), the time-averaged temperatures measured at T1, T2, and T3 were 30.4°C, 29.8°C, and 30.0°C, respectively, corresponding to a spatial temperature range of approximately 0.6°C across the three measurement locations. At a 40°C setpoint (Fig. 10C), the corresponding mean temperatures were 38.9°C, 38.8°C, and 39.9°C, giving a spatial temperature range of approximately 1.1°C. Thus, the spatial temperature variation across the gel was substantially larger than the 0.03°C temporal standard deviation observed at the feedback-control location during the 30°C experiment (Fig. 9A). The spatial variation also increased modestly at the higher temperature setpoint.

The absolute temperature accuracy was distinct from both temporal stability and spatial temperature variation. The thermistors were individually calibrated against a reference thermometer. The calibration procedure, sensor-specific calibration errors, and reference-instrument accuracy are described in detail in the Supplementary Information (Section 6). The calibration results demonstrate good agreement between the calibrated thermistors and the reference measurements; however, the calibration error should not be interpreted as the absolute measurement uncertainty, which is ultimately constrained by the accuracy of the reference thermometer.

These results demonstrate that ARTGEL provides controlled and reasonably uniform temperature conditions across the gel, while also highlighting that spatial temperature variation is larger than the temporal variation at the feedback-control location.

**Fig. 10:**
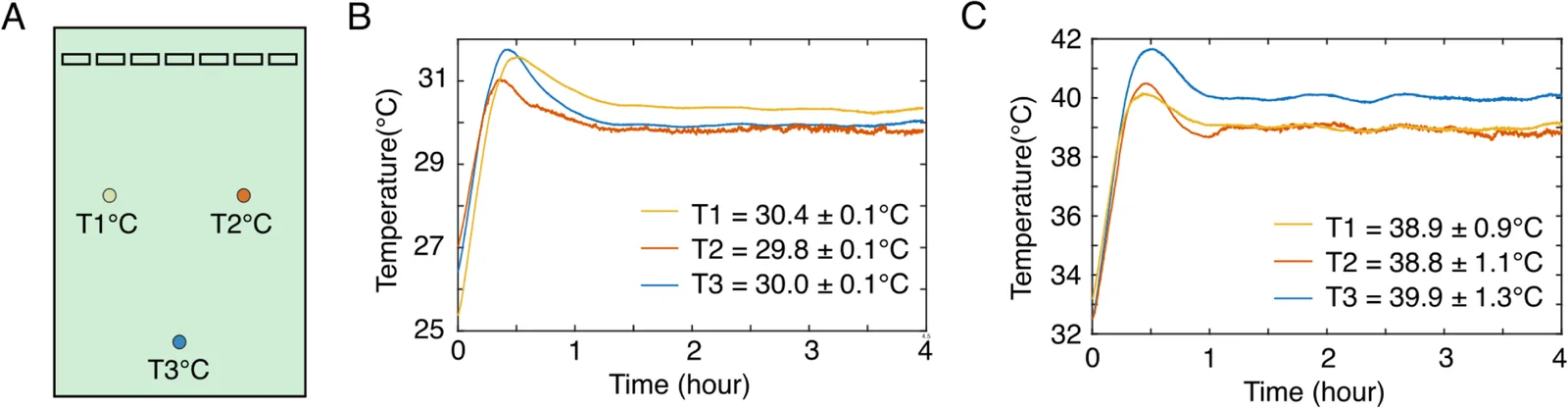
**Spatial temperature distribution in the agarose gel** A. Three locations in the gel where temperature was measured simultaneously, labeled T1, T2, and T3. B. Temperature as a function of time during active temperature regulation at a set temperature of 30°C. C. Temperature as a function of time during active temperature regulation at a set temperature of 40°C. The values reported for T1–T3 represent the mean ± standard deviation of the temperature measured at each location over the plotted period. The difference between the mean temperatures at T1–T3 represents the spatial temperature variation across the gel.

### 7.4 Electrical stability during extended electrophoresis

To evaluate the effectiveness of automated electrode-wiping during long-duration electrophoresis, we performed 24-hour runs with and without active wiping. Here we used a total of 7.2 L of circulating running buffer and replaced external buffer after 12 h. Under high-salt conditions (20 mM MgCl_2_), significant electrochemical deposition on the electrode occurred, increasing its resistance. At the constant applied voltage used for electrophoresis, an increase in this resistance reduces the total current and, according to the series-resistance model described in SI Section 1, reduces the fraction of the applied voltage appearing across the gel.

To quantify this effect, we monitored the electrophoresis current using the programmable electrophoresis power supply as a proxy for electrode condition and system stability. Without electrode wiping, the current decreased substantially from approximately 140 mA to 80 mA over 24 h (Fig. 11A), indicating progressive electrode fouling. In contrast, with periodic automated wiping, the current remained stable at 139.5 ± 2.5 mA throughout the experiment (Fig. 11B). This stability demonstrates that the wiping module effectively mitigates electrode fouling and helps maintain a stable electrophoresis current during extended operation. The voltage drop across the gel was not measured independently in these experiments. Under the series-resistance representation of the electrophoresis circuit, the gel voltage is given by $V_{g}=IR_{g}$, where $R_{g}$ is the gel resistance. Thus, at a fixed applied voltage, maintaining a stable electrophoresis current is consistent with a relatively stable voltage drop across the gel if the gel resistance remains approximately constant. The automated wiping mechanism therefore helps preserve electrical operating conditions by limiting changes in electrode resistance.

Because the voltage drop across the gel was not measured independently, we further evaluated the migration velocities of the monomer and dimer bands, shown in Fig. 12, because electrophoretic velocity is expected to be proportional to the voltage drop across the gel for constant electrophoretic mobility. We observed that the migration velocities remained within 5% over the duration of approximately 24.7 h (SI section 8). The observed changes in velocity therefore provided supporting evidence of voltage stability across the gel.

The total current during electrophoresis can also be influenced by temperature, electrolyte concentration and composition, and electrode polarization. In ARTGEL, active temperature regulation minimizes temperature-dependent changes, continuous buffer circulation helps reduce changes in bulk buffer composition, and automated electrode wiping mitigates progressive changes at the electrode/electrolyte interface. Nevertheless, these factors cannot be completely excluded or independently quantified from the total-current and migration-velocity measurements. Therefore, the current and migration data should be interpreted as indirect evidence for the stability of the voltage drop across the gel rather than as a direct measurement of the gel voltage or electric field. Direct measurement of the voltage between the boundaries of the gel would provide a more rigorous characterization and is an important direction for future validation.

**Fig. 11:**
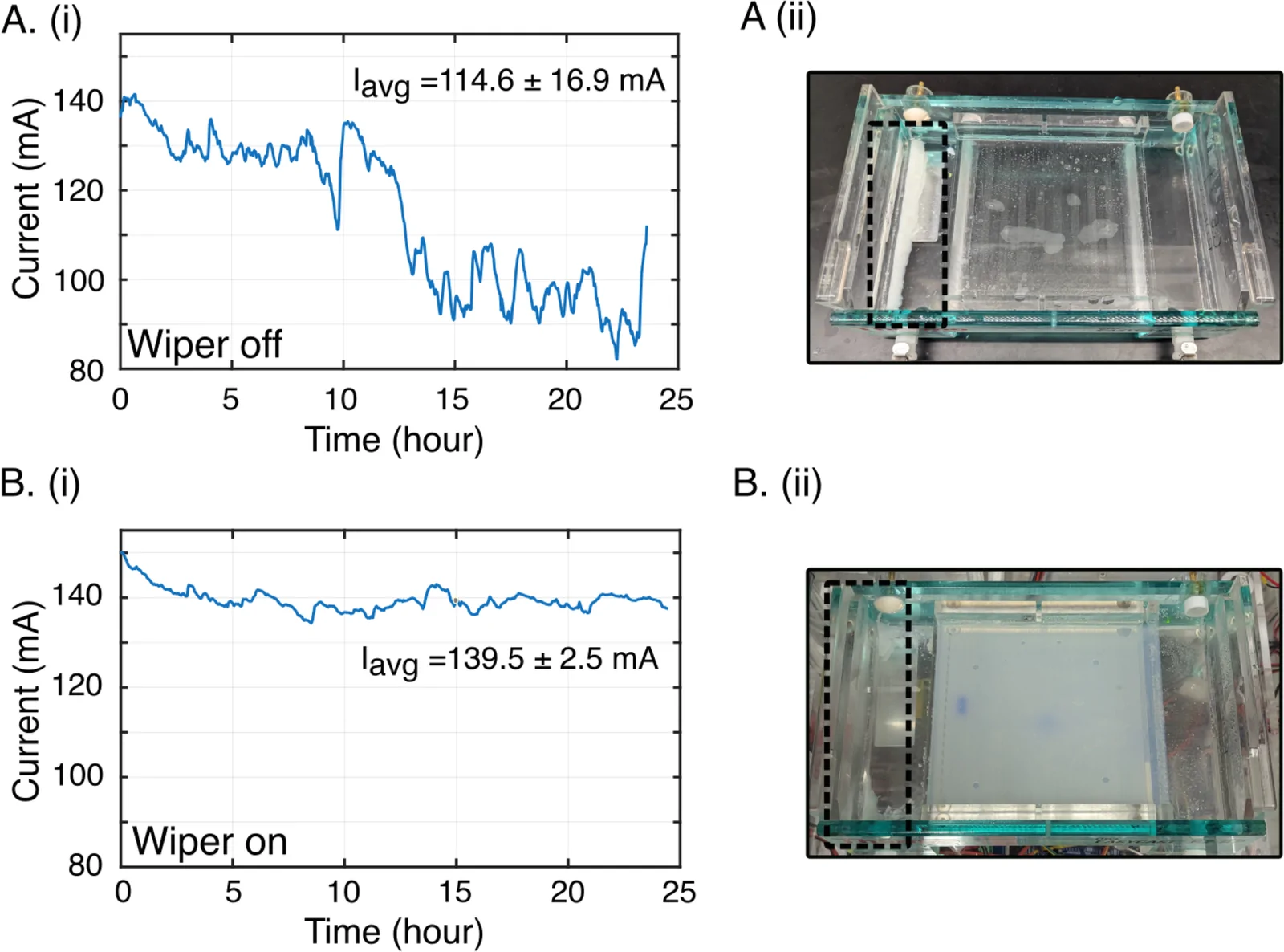
**Wiping performance during gel electrophoresis.** Current as a function of time measured in the absence of wiper (A) and during active wiping (B). Electrochemical deposition ($\text{Mg}(\text{OH}){}_{\text{2}}$) on the negative electrode after electrophoresis, indicated by the dotted rectangular box.

### 7.5 Experiment to quantify reversible dimerization in agarose gel using ARTGEL

Finally, we demonstrated the feasibility of extracting in-gel binding affinity and kinetic parameters for the DNA origami dimerization reaction using ARTGEL. Control samples and reactive samples were first pre-equilibrated at 28°C and then loaded into adjacent lanes of a 1.5% agarose gel maintained at the same temperature (Fig. 12A). Additionally, this electrophoresis experiment was performed at the same temperature as complementary bulk light scattering experiments in the same buffer at thermodynamic equilibrium without the gel, so the resulting in-gel parameters can be compared directly with bulk measurements to determine how confinement and migration in the porous matrix modify reversible self-assembly.

To capture the temporal evolution of the reaction, samples were loaded sequentially at multiple time points during a single electrophoresis run, resulting in five distinct datasets. The earliest loaded samples migrated for approximately 24 h 45 min, while the final set migrated for only 45 min. Throughout the experiment, both gel and buffer temperatures were actively regulated, and the electrophoresis current was continuously monitored to ensure stable operating conditions. Here we used a total of 7.2 L circulating buffer and replaced external buffer after 12 h.

After electrophoresis, the gel was scanned and band intensity profiles were extracted (Fig. 12B). The control samples—consisting of permanent monomers and dimers—separated into two well-resolved peaks, reflecting their distinct electrophoretic mobilities. In contrast, the reactive samples exhibited a continuous intensity distribution between these peaks. This smearing arose from ongoing monomer–dimer interconversion during migration: monomers associated into dimers and slowed down, while dimers dissociated into monomers and accelerated. The reactive dimer peak always contains some monomer and hence on average runs a bit faster than the control dimer peak. Likewise, the reactive monomer peak always contains some dimer and on average runs a bit slower than the control monomer peak. This is clearly visible in Figs. 12A and B by examining the position of the control and reactive bands in side-by-side comparisons at the same time points. Additionally, Figs. 12A and B show that both reactive peaks become skewed and develop characteristic shoulders. The reactive monomer peak becomes skewed only on the “slow” side, widening toward the dimer peak and the reactive dimer peak becomes skewed on the “fast” side, toward the monomer peak.

To analyze the evolution of these asymmetric peak profiles across time and to estimate in-gel kinetic rates and an equilibrium parameter for the gel environment, we simultaneously fit all the concentration profiles to a set of reaction–diffusion–advection equations, details of which will be presented in a separate publication. To enhance the sensitivity of the fit, it is important to run the experiments long enough so that the monomer and dimer peaks are separated by several times their width. To do so in this case, the electrophoresis runs need to be at least 24 h long, justifying the need to develop an instrument with the capabilities of ARTGEL.

An additional advantage of the electrophoretic approach is that the gel resolves the distribution of species present during the reaction. In contrast to bulk-averaged methods such as static and dynamic light scattering, which report ensemble-averaged quantities, ARTGEL reveals whether the sample contains only the assumed reacting species or also additional bands arising from side products, aggregates, or intermediates.

In the present study, this analysis is used to demonstrate the feasibility of extracting in-gel kinetic and equilibrium parameters from the evolving band profiles. A complete and general quantitative treatment of reversible association in gels, including systematic parameter-estimation, sensitivity, and identifiability analyses, is beyond the scope of the present instrumentation-focused study and will be developed separately.

**Fig. 12:**
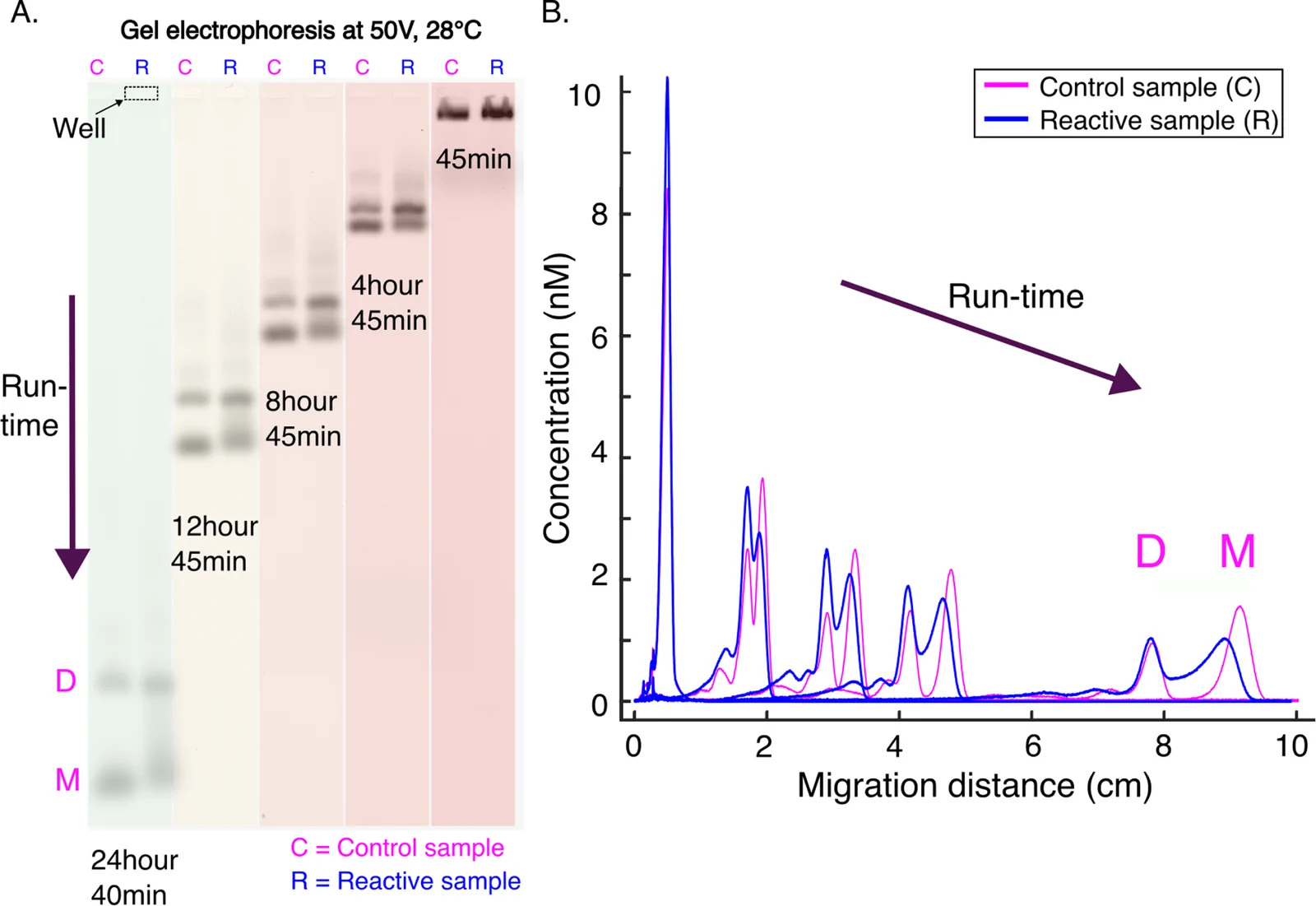
**Gel electrophoresis experiment to extract in-gel kinetic and thermodynamic parameters for DNA origami dimerization using ARTGEL.** (A) DNA origami control and reactive samples were loaded in 1.5% agarose gel at different time points while gel temperature was maintained at 28°C. The loading wells were visible at the top of the image as faint, light rectangular regions directly under the labels “C” and “R”. The gel was scanned using a GE Typhoon FLA 9500 Imager. (B) The measured fluorescent intensity, which was proportional to the concentration as a function of position, of the control and reactive samples.

### 7.6 Safety considerations

The operation of ARTGEL involves electrochemical reactions, electrical power, thermal control, and circulating aqueous buffer and therefore requires appropriate laboratory safety precautions. During electrophoresis, electrochemical reactions occur at the electrodes. In the chloride-containing buffer used in ARTGEL, anodic oxidation of chloride can potentially produce chlorine or other chlorine-containing oxidants, while cathodic reduction of water can generate hydrogen gas. Gas production was not directly quantified in this study. Accordingly, ARTGEL was operated in a well-ventilated laboratory environment. Potential ignition sources were kept away from the electrophoresis apparatus during operation.

Because ARTGEL combines circulating buffer with electrical components, fluidic and electrical safety measures were incorporated into the system design. The electrophoresis chamber, buffer reservoir, tubing, and fluidic connections were inspected before each experiment for leaks, and the circulation tubing was purged before operation to ensure continuous flow. Moat-like channels incorporated into the top surface of the electronic enclosure direct leaked liquid away from the electronic components. Electrical components were enclosed and separated from the fluidic pathways. The electrophoresis and temperature-control systems were de-energized before handling the gel, temperature sensors, electrode-wiper assembly, or samples.

Temperature was continuously monitored during operation. In addition to software-based temperature control, a hardware-level thermal safety switch rated at 65°C was attached to the thermoelectric heat-spreading plate to provide independent protection against excessive heating. The temperature-control system and electrophoresis power supply have independent power switches, allowing either system to be de-energized in the event of abnormal operation, leakage, or electrical malfunction.

Following electrophoresis, used buffer and material deposited on the electrodes were handled using appropriate laboratory precautions and were disposed of according to applicable institutional environmental health and safety procedures and local regulations.

As a future safety enhancement, ARTGEL could incorporate leak and flow sensors around the electrophoresis chamber and electronic enclosure. These sensors could be coupled to visual or audible alarms and, for critical faults, to an automated shutdown routine that de-energizes the electrophoresis and temperature-control systems during unattended long-duration experiments.

### 7.7 Discussion

The thermistors were electrically insulated and housed within nickel probes, whose electrical and thermal conductive properties may locally perturb the electric and temperature fields. The probes were positioned away from the sample-loading and migration regions and from the buffer inlet and outlet pathways to minimize their potential influence on the measurements and bulk buffer circulation.

The three embedded thermistors characterize temperature variation at discrete locations but do not provide a complete spatial map of the gel temperature. Spatially resolved infrared thermography could provide complementary validation [39]. However, implementation in the present ARTGEL configuration would require modification of the chamber to provide an appropriate optical path to the gel, which is immersed in circulating buffer and enclosed within the electrophoresis chamber and thermoelectric module. Quantitative infrared measurements would also require calibration of the transmission and emissivity of the intervening materials. Such measurements could therefore provide a useful complementary approach for future characterization of the spatial temperature field.

The spatial distribution of the electric field is another factor that may influence electrophoretic measurements. ARTGEL retains the electrode and gel geometry of a commercially manufactured Thermo Scientific Owl EasyCast horizontal electrophoresis system rather than introducing a custom electrophoresis geometry. This standardized geometry provides the underlying electrophoretic configuration used in the present experiments. Nevertheless, local electric-field perturbations associated with the wells, gel boundaries, temperature probes, and automated electrode-wiping mechanism cannot be excluded. Direct potential measurements or numerical simulations could therefore provide a useful future approach for quantitatively characterizing the spatial electric-field distribution and assessing local perturbations introduced by the ARTGEL components.

### 7.8 Conclusion

In this work, we developed ARTGEL, a temperature-regulated agarose gel electrophoresis platform that enables long-duration experiments under independently controlled thermal and electrical conditions. By maintaining a constant gel temperature and using automated electrode wiping to mitigate electrode fouling and maintain stable electrophoresis current, ARTGEL creates a well-defined in-gel environment for quantitative electrophoresis of reactive systems. These capabilities allow electrophoresis to be performed at the same temperature as complementary bulk measurements while preserving the experimental control needed to interpret reaction during migration.

Using DNA origami assemblies, we show that ARTGEL preserves distinct temperature-dependent association states, provides stable gel temperatures over many hours, maintains relatively small spatial temperature variations within the gel, and maintains stable electrophoresis current for runs exceeding 24 h. Under these conditions, reactive samples develop characteristic asymmetric profiles and shoulders indicative of ongoing association and dissociation during migration. These features can be analyzed quantitatively to extract in-gel kinetic and thermodynamic parameters rather than assuming that the gel is a passive readout of the pre-equilibrated bulk solution.

ARTGEL therefore extends the role of gel electrophoresis beyond qualitative separation. It provides a practical and open-source platform for studying how confinement in porous media influences reversible biomolecular association, for comparing in-gel behavior with bulk measurements performed under matched temperature conditions, and for spatially resolving the distributions of species present before loading as well as those generated during migration and reaction, including fragments, side products, aggregates, or additional association states. More broadly, ARTGEL makes gel electrophoresis useful as a quantitative tool for reactive transport, reversible self-assembly, and temperature-sensitive nanoscale systems in gels.

ARTGEL is implemented as a modular platform using widely available components, with the hardware designs, firmware, control software, and bill of materials shared to facilitate adaptation and further optimization. Although some components presently contribute substantially to the overall cost, the design is flexible and can be further standardized or simplified for other applications. Future extensions could integrate real-time fluorescence imaging [39] and direct measurements of the electric-field distribution to provide more comprehensive characterization of coupled reaction, transport, and thermal behavior in gels.

## CRediT authorship contribution statement

**Rupam Saha:** Writing – review & editing, Writing – original draft, Validation, Software, Methodology, Investigation, Formal analysis, Data curation. **Seth Fraden:** Writing – review & editing, Supervision, Resources, Investigation, Funding acquisition, Conceptualization.

## Ethics statements

We confirm that our work does not involve any animal or human experiments.

## Declaration of competing interest

The authors declare that they have no known competing financial interests or personal relationships that could have appeared to influence the work reported in this paper.

## Figures and Tables

**Table:** 

**Abbreviation**	**Definition**

ARTGEL	Actively Regulated Temperature Gel Electrophoresis
EMSA	Electrophoretic Mobility Shift Assay
GUI	Graphical User Interface
PI	Proportional–Integral
PWM	Pulse-Width Modulation
AWG	American Wire Gauge
DC	Direct Current
NPSM	National Pipe Straight Mechanical
NTC	Negative Temperature Coefficient

## Appendix A Supplementary data

Video S1

MMC S2
