# Supplementary material for: ARTGEL: A temperature-regulated gel electrophoresis platform for quantitative studies of reversible association
Source: HardwareX. 2026 Oct 17;28:e00847. doi: 10.1016/j.ohx.2026.e00847 (PMC13722569; doi:10.1016/j.ohx.2026.e00847)
Supplement: Supplementary file 1 [file mmc2.pdf]

Supplementary information for "ARTGEL: A temperature-regulated gel electrophoresis platform for quantitative studies of reversible association"

Authors
*Rupam Saha, Seth Fraden**

Affiliations
Martin A. Fisher School of Physics, Brandeis University, Waltham, Massachusetts 02453, USA

Corresponding author's email address
fraden@brandeis.edu

1. Two resistors model for electrode fouling

The electrophoresis system can be modeled as two electrical resistances connected in series: a constant gel resistance, R_g, and a time-dependent electrode resistance, $R_e(t)$, combining both electrodes, which increases due to salt deposition at the electrode surface. The total circuit resistance is therefore

$$R_{\text{tot}}(t) = R_g + R_e(t). \tag{1}$$

Under constant applied voltage, V_{app}, the electrophoresis current is given by Ohm's law:

$$I(t) = \frac{V_{\text{app}}}{R_g + R_e(t)}. \tag{2}$$

As salt accumulates on the electrode, $R_e(t)$ increases, resulting in a reduction of the electrophoresis current.

The voltage drop across the gel, which determines the electric field experienced by the migrating species, is

$$V_g(t) = I(t)\, R_g = V_{\text{app}}\, \frac{R_g}{R_g + R_e(t)}. \tag{3}$$

For a gel of length L, the electric field within the gel is

$$E(t) = \frac{V_g(t)}{L} = \frac{V_{\text{app}}}{L}\, \frac{R_g}{R_g + R_e(t)}. \tag{4}$$

The electrophoretic velocity is proportional to the electric field,

$$v(t) = \mu\, E(t), \tag{5}$$

where μ is the electrophoretic mobility. Substituting for $E(t)$ yields

$$v(t) = \mu\, \frac{V_{\text{app}}}{L}\, \frac{R_g}{R_g + R_e(t)}. \tag{6}$$

An increase in electrode resistance due to salt deposition therefore leads to a decrease in current, electric field, and electrophoretic velocity over time. Active electrode wiping suppresses the growth of $R_e(t)$, thereby reducing changes in total circuit resistance and helping maintain a stable electrophoresis current. Under the assumption that the gel resistance and electrophoretic mobility remain approximately constant, this is consistent with relatively stable electric-field strength and electrophoretic velocity during long-duration electrophoresis.

2. Electrochemical Depletion of $MgCl_2$ During Electrophoresis

In electrophoresis experiments, sustained current flow through the buffer leads to electrochemical reactions at the electrodes that can alter the buffer composition. In the aqueous $MgCl_2$-containing buffer, possible electrode reactions include chloride oxidation at the anode and water reduction at the cathode:

$$Anode(+) : 2\,\mathrm{Cl}^- \rightarrow \mathrm{Cl}_2(g) + 2\mathrm{e}^- (\text{Chloride ions are oxidized to chlorine gas}) \tag{7}$$

$$Cathode(-) : 2\,\mathrm{H}_2\mathrm{O} + 2\mathrm{e}^- \rightarrow \mathrm{H}_2(g) + 2\,\mathrm{OH}^-(aq) (\text{Water is reduced to hydrogen and hydroxide ions}) \tag{8}$$

At the cathode, the produced OH^- ions react with Mg^{2+} ions to form insoluble magnesium hydroxide:

$$\mathrm{Mg}^{2+} + 2\,\mathrm{OH}^- \rightarrow \mathrm{Mg(OH)}_2 \tag{9}$$

The reactions above represent possible electrochemical pathways. The products and relative contributions of the individual electrode reactions were not directly identified in the present experiments. Therefore, the calculation below is intended as a Faradaic upper-bound estimate of the amount of $MgCl_2$ that could be removed from the system based on the measured electrophoresis current.

Our electrophoresis gel box contains approximately 0.8 L of 20 mM $MgCl_2$ solution including 0.2 L gel and 0.6 L running buffer. During electrophoresis, $MgCl_2$ steadily depletes from the system. We calculate the rate of salt depletion below.

The total electric charge passed through the process is given by:

$$Q = I \cdot t \tag{10}$$

where I is the applied current (A) and t is the duration of electrophoresis (s). For an average current of $I = 0.13$ A applied over $t = 3600$ s (1 hour), the total charge is:

$$Q = 0.13 \times 3600 = 468 \text{ C} \tag{11}$$

Using Faraday's constant ($F = 96485$ C/mol), the number of moles of electrons transferred is:

$$n_e = \frac{Q}{F} = \frac{468}{96485} \approx 4.85 \times 10^{-3} \text{ mol} \tag{12}$$

Since each $MgCl_2$ unit provides two chloride ions, the number of moles of $MgCl_2$ depleted is:

$$n\mathrm{MgCl}_2 = \frac{n_e}{2} = \frac{4.85 \times 10^{-3}}{2} \approx 2.43 \times 10^{-3} \text{ mol} \tag{13}$$

The electrophoresis chamber contains approximately 0.6 L of free running buffer and approximately 0.2 L of agarose gel, giving a total $MgCl_2$-containing volume of approximately 0.8 L. With an initial $MgCl_2$ concentration of 20 mM, the initial amount of $MgCl_2$ is:

$$n_0 = 0.020 \times 0.8 = 0.016 \text{ mol} \tag{14}$$

Thus, the fractional depletion per hour is:

$$\frac{n_{\mathrm{MgCl}_2}}{n_0} \approx \frac{2.43 \times 10^{-3}}{0.016} \approx 0.15 \tag{15}$$

indicating that approximately 15% of the $MgCl_2$ is depleted per hour under these conditions. At this rate of depletion, no Mg^{2+} would be left after a 24 hour run. To reduce the percentage change in Mg^{2+} concentration over 24 hours, we circulate a large volume of heated buffer stored in the external buffer tank through the electrophoresis chamber. The circulating volume of buffer depends on the duration of experiment. Typically, For a 24-h experiment, the system contains approximately 7.4 L of $MgCl_2$-containing material, comprising 0.6 L of free buffer in the electrophoresis chamber, approximately 0.2 L of gel containing buffer at the same nominal $MgCl_2$ concentration, and 6.6 L of buffer in the external reservoir which is replaced after 12 h with freshly prepared buffer.

The initial amount of $MgCl_2$ in the system is

$$n_0 = 0.020 \times 7.4 = 0.148 \text{ mol} \tag{16}$$

Thus, the fractional depletion per hour is:

$$\frac{n_{\mathrm{MgCl_2}}}{n_0} \approx \frac{2.43 \times 10^{-3}}{0.148} \approx 0.016 \tag{17}$$

Thus, the calculated $MgCl_2$ removal corresponds to approximately 1.6% of the initial $MgCl_2$ inventory per hour. Because the external reservoir-buffer is replaced after approximately 12 h during the long-duration experiments, the estimated depletion does not accumulate continuously over the full 24-h period. Using the total $MgCl_2$-containing volume of approximately 7.4 L, the Faraday-law estimate corresponds to a fractional depletion of approximately 1.6% per hour, or approximately 19% over a 12-h interval at an average current of 0.13 A.

The calculation above provides a first-order theoretical estimate of the extent of $MgCl_2$ depletion based on the measured average electrophoresis current and assumes that the charge passed through the system is directly associated with the electrochemical reactions described above. It therefore provides an estimate of the scale of depletion rather than a direct measurement of the Mg^{2+} concentration in the circulating buffer. Direct measurements of conductivity, pH, or Mg^{2+} concentration during prolonged electrophoresis were not performed in the present study but would provide a experimental validation of the aforementioned estimate.

3. PI Control and Controller Tuning

We regulate the gel temperature using a proportional–integral (PI) feedback control loop that adjusts the pulse-width modulation (PWM) signal in response to the measured temperature error. The continuous-time PI control law is

$$u(t) = K_p e(t) + K_i \int_0^t e(\tau)\, d\tau, \tag{18}$$

where

$$e(t) = T_{\mathrm{set}} - T(t), \tag{19}$$

with $u(t)$ representing the control signal, K_p and K_i the proportional and integral gains, T_{set} the target temperature, and $T(t)$ the measured temperature.

The PI controller is implemented digitally on the Arduino. The controller does not use a fixed sampling period; instead, the elapsed time between successive control-loop iterations, Δt_n, is used for numerical integration. At control step n,

$$e[n] = T_{\mathrm{set}} - T[n], \tag{20}$$

$$I[n] = I[n-1] + e[n]\Delta t_n, \tag{21}$$

and

$$u[n] = K_p e[n] + K_i I[n]. \tag{22}$$

Thus, the effective control-loop interval is determined by the execution time of the Arduino loop rather than by a fixed programmed delay or timer. The integral state can be reset using the controller reset command, which sets the accumulated integral term to zero.

The signed control signal is converted to the PWM magnitude and heating or cooling direction according to

$$PWM[n] = \min(|u[n]|, 255), \tag{23}$$

where the PWM magnitude is limited to 0–255 and the sign of $u[n]$ determines the polarity applied to the thermoelectric module through the H-bridge. Thus, the effective signed actuator command ranges from -255 to $+255$. No explicit integral anti-windup mechanism is implemented; when the actuator reaches its PWM limit, the integral term continues to accumulate.

The controller does not currently include automated software-based fault-detection or fault-handling. In the event of a component failure or system malfunction, the affected experiment is excluded from analysis and repeated. Independent hardware-level thermal protection is provided by the thermal safety switch described in the main manuscript.

3.1 Experimental setup

We prepared 1.5% agarose gel and a running buffer of volume 0.6 L at 20 mM $MgCl_2$ for the electrophoresis chamber. We prepared an additional 6.6 L buffer to be stored in the external reservoir and heated it to 32 °C. After the buffer temperature stabilized at the setpoint, buffer circulation between the external reservoir and the electrophoresis chamber was initiated at a flow rate of 30 mL/min with the insulation enabled. An electrophoresis voltage of 50 V was then applied, and the gel temperature was continuously monitored. Once the gel temperature reached a steady-state plateau, the following experiments were performed. Throughout all subsequent experiments, buffer circulation, insulation, and the applied electrophoresis voltage were maintained continuously.

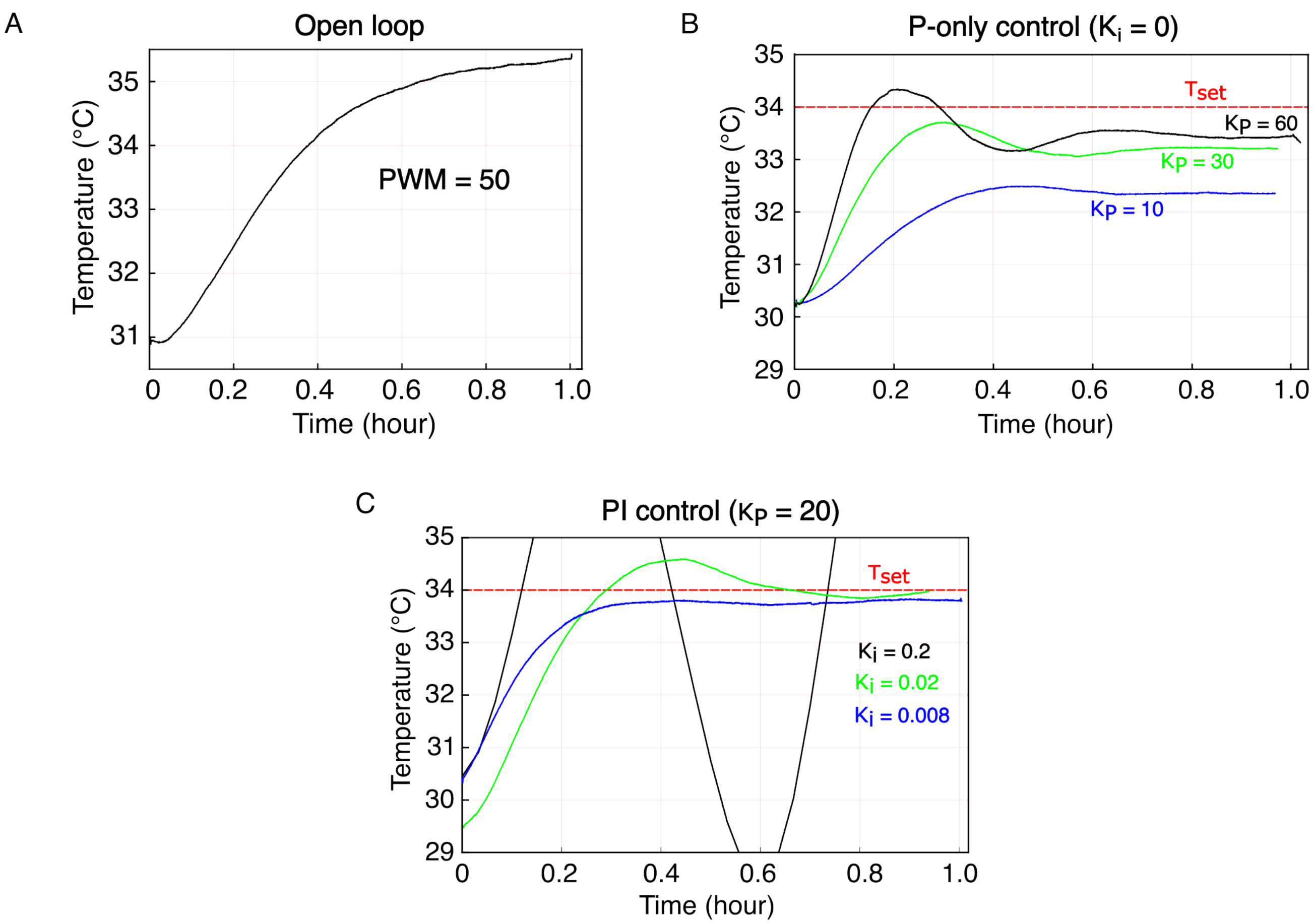

Figure S1: **Parameter sweep for proportional-integral control of the gel.** (A) Manual or open loop control. (B) K_P sweep (C) K_i sweep when $K_p = 20$

3.2 Open-loop thermal response

To characterize the intrinsic thermal dynamics of the system, we first performed an open-loop experiment (SI Fig. S1A). A constant PWM value of 50 was applied for one hour, resulting in an increase in gel temperature from 31°C to 35°C, after which the system approached a steady-state plateau. This slow response indicates a large thermal time constant on the order of tens of minutes, reflecting the combined heat capacity of the gel and surrounding buffer.

3.3 Proportional control (K_p sweep)

We next evaluated proportional (P) control by setting $K_i = 0$ and varying K_p (SI Fig. S1B). In this regime, the control signal is directly proportional to the instantaneous temperature error. While this improves response speed, it inherently produces a steady-state offset (droop), since a nonzero error is required to sustain the control action.

At low gain ($K_p = 10$), the system approaches the setpoint (34°C) slowly and exhibits a steady-state error of 1.8°C. Increasing the gain to $K_p = 30$ reduces the steady-state error to 0.8°C and accelerates convergence. At high gain ($K_p = 60$), the system responds rapidly but exhibits oscillatory behavior due to overcorrection in the presence of thermal delay, settling with a residual error of approximately 0.6°C. These results demonstrate the trade-off between responsiveness and stability, with $K_p \approx 20$–30 providing a practical compromise.

3.4 Proportional–integral control (K_i sweep)

To eliminate steady-state error, we introduced an integral term, which accumulates the error over time and drives the system toward the exact setpoint. However, the integral gain must be carefully tuned for slow thermal systems.

We fixed $K_p = 20$ and varied K_i (SI Fig. S1C). At high integral gain ($K_i = 0.2$), the system exhibits large-amplitude, long-period oscillations (approximately 30 min) due to rapid accumulation of the integral term, leading to overshoot and slow recovery. At moderate gain ($K_i = 0.02$), the system achieves stable convergence to the setpoint with minimal oscillation. At low gain ($K_i = 0.008$), convergence is slower and small residual errors persist over extended times.

3.5 Optimal control parameters

Together, these results indicate that stable and efficient temperature regulation is achieved with $K_p \approx 20$ and $K_i \approx 0.02$. Under these conditions, the system reaches a 4°C temperature increase within approximately 30 minutes while maintaining minimal oscillation and negligible steady-state error.

In addition to regulating the gel temperature, the temperature of the buffer in the external reservoir was also controlled using a separate proportional–integral (PI) control loop implemented in the Arduino firmware. The PI parameters for the buffer-heating module were not systematically optimized because the thermal mass of the reservoir can vary with the experimental configuration and buffer volume. Therefore, the reported parameters should be considered operating parameters rather than optimized controller settings.

4. Temperature acquisition and data processing

Temperature was measured using 100 kΩ NTC thermistors with a nominal Beta parameter of 4540 K for the lot used in the experiments. The thermistor signals were acquired using the 10-bit analog-to-digital converter (ADC) of the Arduino. At each acquisition cycle, 5000 consecutive ADC readings were averaged before conversion to temperature, and temperature measurements were recorded at approximately 2 s intervals. For presentation and calculation of the temporal temperature stability shown in Fig. 9, the recorded temperature data were subsequently averaged over 2-min intervals. The mean and standard deviation of these 2-min-averaged measurements were used to report T_{avg}.

5. Use of plugs

We designed and 3D printed well plugs for gels with 1.5 mm thick wells. The wells had dimensions $l \times w \times h = 4 \times 1.5 \times 9$ mm^3 and the plug had dimensions $l \times w \times h = 4 \times 1.5 \times 4$ mm^3 (Figure S2). Each plug features two teeth, allowing it to anchor securely across adjacent wells and improve stability during operation.

Figure S3 shows a representative gel in which control and reactive samples are loaded into adjacent lanes at sequential time points, in which the lanes on the left are loaded at the beginning of the experiment and those on the right are loaded after 6 hours of electrophoresis. During the initial loading step, the wells show no fluorescence, but over time fluorescence from the DNA origami increases in the wells. Our hypothesis is that the fluorescence comes from origami complexed with $Mg(OH)_2$ precipitants. No precipitant forms in the beginning of the experiment. However, as the experiment progresses, the electrode wipers continuously remove $Mg(OH)_2$ from the negative electrode. Some of this material subsequently redeposits into the wells. We hypothesize that the DNA origami loaded late in the run becomes adsorbed to, or physically trapped in this hydrated hydroxide deposit. As a result, the concentration of DNA origami that moves through the gel at late times will be less than DNA origami injected into the gel at early times.

Overall, the use of plugs helps maintain the concentration of origami in the gel during the 24 hour period of gel electrophoresis.

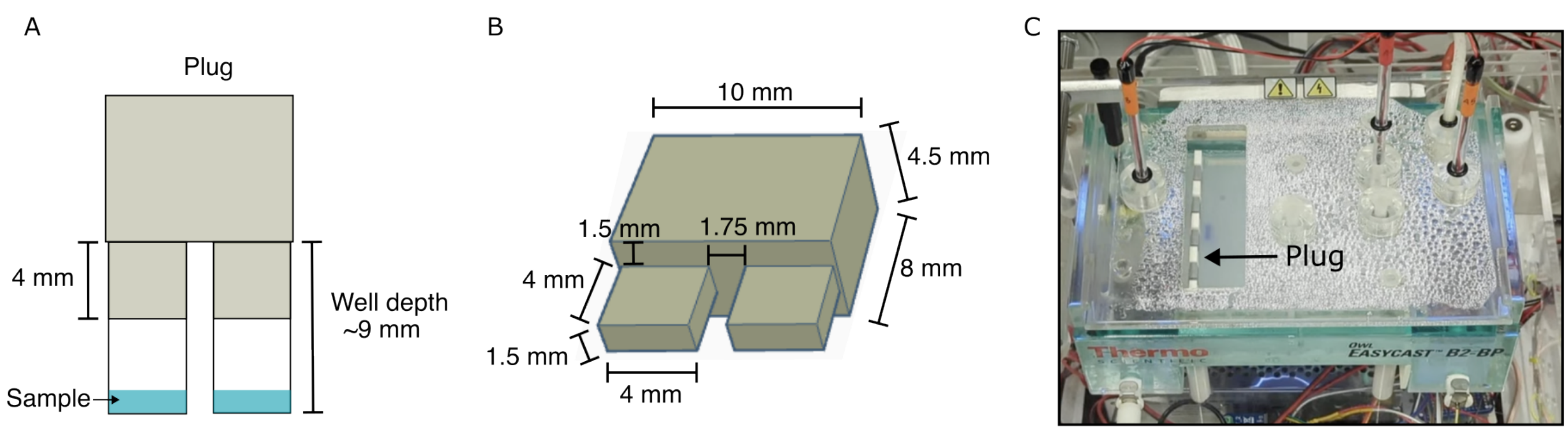

Figure S2: **3D-printed Plug to block the gel wells** (A)A plug with two teeth placed in wells. (B) 3D design of a plug with measurements. (C) An image of a gel where the wells are blocked with plugs.

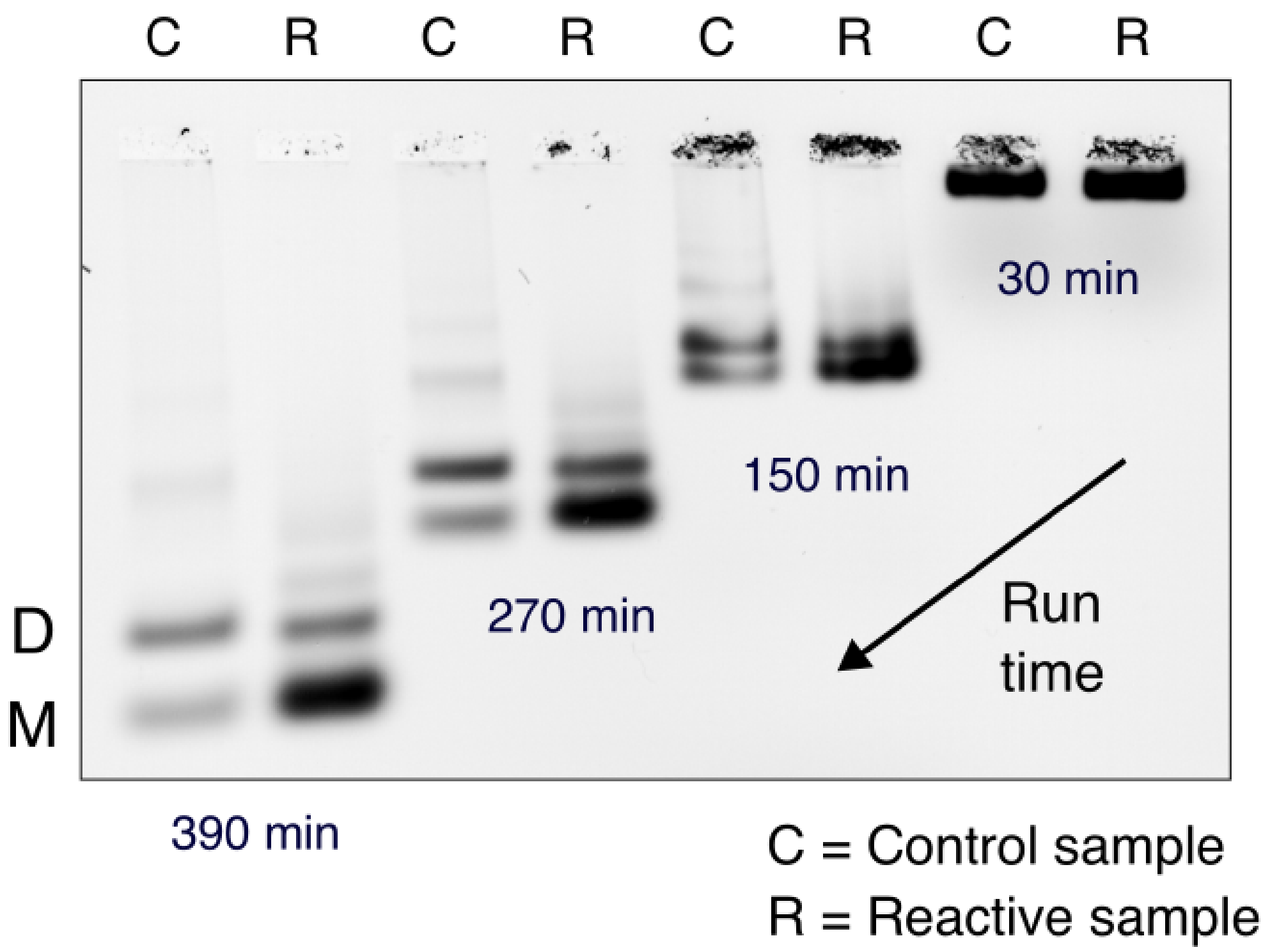

Figure S3: **Gel electrophoresis with open wells (without plugs).** A laser-scanned micrograph of a gel loaded with control and reactive DNA origami samples.

6. Calibration of thermistor

Prior to installation, all thermistors were calibrated in a stirred water bath together with a reference digital K-type thermocouple thermometer over a temperature range 20° C - 44° C. At each temperature condition, the reference K-type thermocouple and all thermistors were simultaneously immersed in the water bath, and temperature data were recorded after the system reached a stable condition.

For each thermistor, the measured divider voltage was first converted to resistance and then to a raw temperature value using the Beta-parameter model for an NTC thermistor:

$$\frac{1}{T} = \frac{1}{T_0} + \frac{1}{\beta} \ln\left(\frac{R}{R_{25}}\right) \tag{24}$$

where $\beta = 4540$ K and $R_{25} = 100$ kΩ. The raw temperature measurements acquired during each calibration condition were averaged separately for each thermistor sensor to obtain one representative temperature value per sensor. Similarly, the equilibrium temperatures measured by the reference thermocouple were averaged for each calibration condition. A linear regression was then performed for each thermistor according to

$$T_m = mT_{\text{ref}} + c \tag{25}$$

where T_m is the raw thermistor-reported temperature, T_{ref} is the thermocouple temperature, and m and c are the sensor-specific slope and intercept, respectively. During subsequent instrument operation, each thermistor reading was corrected using the inverse relation

$$T_r = \frac{T_m - c}{m} \tag{26}$$

where T_r is the calibrated reported temperature. This procedure compensates for sensor-to-sensor variation while retaining a common nominal thermistor model for all sensing channels.

The calibration performance was quantified by comparing the calibrated thermistor temperatures with the corresponding reference-thermocouple temperatures at all the calibration conditions. The mean absolute error was calculated as

$$\text{MAE} = \frac{1}{N} \sum_{i=1}^{N} |T_{r,i} - T_{\text{ref},i}|$$

where N = 4 is the number of calibration conditions. The resulting mean absolute errors for thermistors $A2^1$, $A3^1$, $A4^1$, $A5^1$, and $A0^2$ were 0.08, 0.04, 0.12, 0.06, and 0.10 °C, respectively relative to the reference thermocouple measurements.

The reference K-type thermocouple had a manufacturer-specified accuracy of ±(0.3% of reading + 1°C) which is approximately $\pm 1.06 - 1.14$ °C for a temperature range of $20 - 45$ °C. Thus, the mean absolute error values reported above characterize the agreement of the calibrated thermistors with the reference thermometer over the calibration conditions and should not be interpreted as an independent absolute accuracy of the temperature measurement. The absolute accuracy of the calibrated temperature measurements is ultimately limited by the accuracy of the reference thermometer.

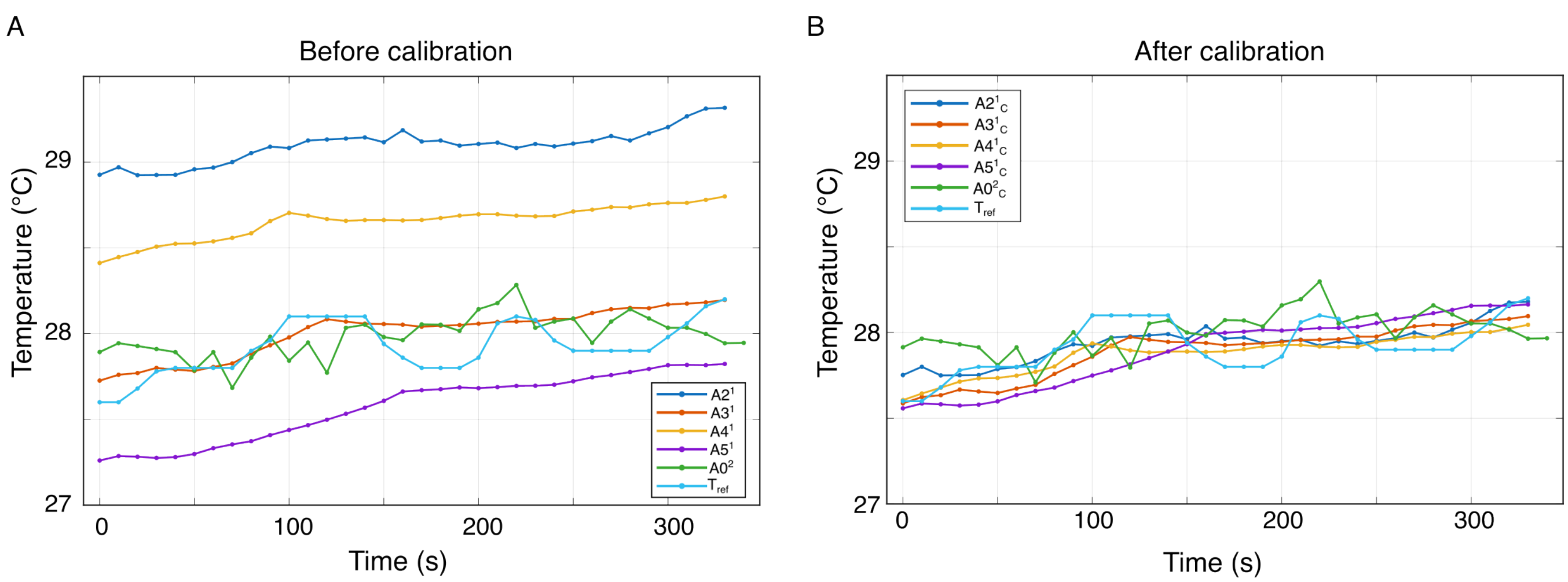

Figure S4: **Calibration of thermistors using a reference thermocouple.** (A) Temperature versus time measured during the calibration experiment by the individual thermistors (see Fig. 4 for thermistor labels) and reference thermocouple (T_{ref}) before application of the sensor-specific linear correction. (B) The same calibration data replotted after application of the corresponding sensor-specific calibration correction, together with the reference thermocouple (T_{ref}) measurement.

7. Measurement of the time required for DNA to exit the gel well

The time required for DNA origami monomers to exit the sample well and enter the agarose gel was experimentally evaluated using gel combs with three different well widths, $W = 0.75$ mm, 1.0 mm and 1.5 mm. The sample wells had dimensions of 4 mm (length) $\times$ W mm (width) $\times$ 9 mm (depth), corresponding to a total well volumes of 27, 36 and 54 µL, respectively. All experiments were performed using 1.5% agarose gels in running buffer containing 20 mM $MgCl_2$, under identical electrophoresis conditions at an applied voltage of 50 V. For each experiment, 6 µL of sample was loaded into the well, consisting of 5 µL of DNA origami sample mixed with 1 µL of loading dye.

Following gel preparation, identical monomer samples were loaded sequentially from left to right across the gel. This staggered loading procedure allowed different effective electrophoresis times within a single run, such that the first loaded sample migrated for 30 min, whereas the final sample migrated for only 2 min before the run was terminated.

At the completion of electrophoresis, each sample well was thoroughly rinsed to remove any residual DNA remaining inside the well. The gel was then imaged, and intensity profiles extracted from the scanned image after background subtraction. For each lane, the area under the migrated DNA peak was determined. The largest peak area measured in the experiment was taken as the reference corresponding to complete sample release. A mass fraction was then defined as the ratio of the peak area for a given lane to this maximum value, representing the fraction of DNA that had exited the well.

The results indicate that approximately 20 min were required for nearly complete release of the DNA sample from the well under the tested conditions. Initially, it was hypothesized that narrower wells would shorten the exit time by reducing the distance required for DNA transport within the well. However, only a weak dependence on well width was observed. This suggests that the dominant transport resistance arises at the well–gel interface rather than within the well itself. Our physical picture is that DNA molecules are transported rapidly within the liquid-filled well and accumulate transiently at the gel boundary, while the slower migration into the agarose matrix limits the overall entry rate. Consequently, the release kinetics are largely insensitive to well width over the tested range. These observations further suggest that the gel-entry time is more strongly governed by parameters such as the applied voltage and agarose concentration, than well width.

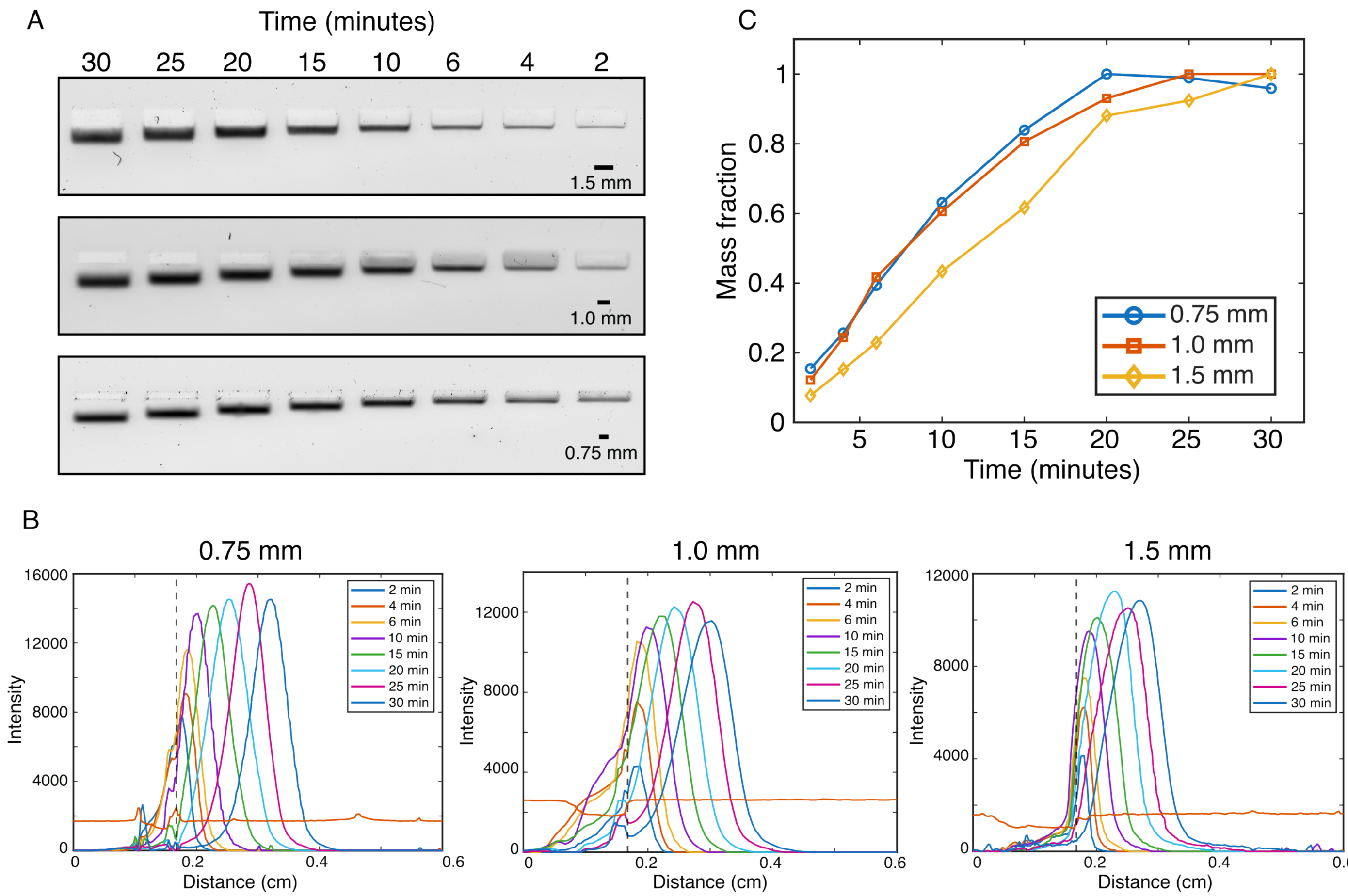

Figure S5: **Gel electrophoresis with varying well width.** (A) Gel electrophoresis (1.5% agarose gels, 50 V) with wells of three different widths: 1.5 mm, 1.0 mm, and 0.75 mm (top to bottom). The same volume of passivated monomer sample, 6 microliter, is loaded in each well at different time points. The total run time of sample at each well is recorded. (B) Background-subtracted intensity profiles of the corresponding DNA bands. The distance axis refers to the vertical axis along the electrophoresis direction of DNA including the well and the band. The dotted vertical line indicates the boundary of the well, beyond which DNA has entered the agarose gel. (C) Mass fraction as a function of run time, where the mass fraction is defined as the ratio of the area under an individual intensity profile to the maximum measured area. For this analysis, only the area beyond the dotted line in panel (B) was included. It takes about 15 minutes for 80% of the origami in the 0.75 mm and 1.0 mm wells to enter the gel and about 20 minutes for 80% of the origami in the 1.5 mm well to enter the gel.

8. Stability of voltage across the gel during extended electrophoresis

We evaluated long-duration electrophoretic performance by continuously monitoring the electrophoresis current and analyzing monomer and dimer migration velocities during an extended gel electrophoresis experiment (Fig. S6A). Electrophoresis was performed at an applied voltage of 50 V and a temperature setpoint of 28 °C using 20 mM $MgCl_2$ running buffer. A total of 7.2 L of running buffer was used, including 6.6 L contained in the external reservoir. As shown in Fig. S6B, the gel temperature was regulated at 28 ± 0.1 °C throughout the experiment. The corresponding buffer temperature was maintained at 28 ± 0.95 °C (Fig. S6C). The transient change in buffer temperature reflects replacement of the external buffer after approximately 12 h.

The electrophoresis current was monitored continuously throughout the experiment (Fig. S6D). The current initially remained near 140 mA and gradually decreased to approximately 130 mA. This gradual decrease indicates that electrode fouling was not completely eliminated during this particular extended experiment. Although the automated electrode wiper was operated during the experiment, electrode fouling was not completely eliminated. The resulting increase in electrode resistance caused the total circuit resistance to increase and the electrophoresis current to decrease under constant applied voltage.

We next analyzed the migration of monomer and dimer DNA origami bands at four electrophoresis times spanning approximately 24.7 h (Fig. S6E). Band positions were determined by Gaussian fitting of background-corrected fluorescence intensity profiles, and migration distances were calculated relative to the downstream edge of the loading well (Fig. S6A). The monomer migration velocity ranged from 9.42×10^{-5} to 9.86×10^{-5} cm/s, corresponding to an increase of approximately 4.7% over the experimental duration. The dimer migration velocity ranged from 8.13×10^{-5} to 8.39×10^{-5} cm/s, corresponding to an increase of approximately 3.2%.

The samples were loaded sequentially such that the sample intended to undergo the longest electrophoresis duration was loaded first, followed by the samples intended for shorter durations. Consequently, the longest-running sample experienced the relatively higher initial current, when the electrodes were less fouled, whereas the samples loaded later experienced progressively lower current as electrode resistance increased. According to the series-resistance model of the electrophoresis circuit, an increase in electrode/interfacial resistance at fixed applied voltage causes a reduction in the current and, consequently, a reduction in the voltage drop across the gel. Because electrophoretic migration velocity is proportional to the electric field for a given electrophoretic mobility, the observed increase in migration velocity with increasing electrophoresis time is consistent with changes in the voltage drop across the gel arising from changes in the electrode/interfacial resistance.

The migration-velocity measurements therefore provide supporting evidence for the stability of the voltage across the gel. If the electrophoretic mobility of each species is assumed to remain approximately constant over the duration of the experiment, the relative change in migration velocity provides an estimate of the corresponding relative change in the voltage drop across the gel. Under this assumption, the monomer and dimer velocities changed by approximately 4.7% and 3.2%, respectively. Thus, the velocity measurements indicate that the voltage drop across the gel changed by no more than approximately 5% over the approximately 24-h experiment. This analysis should be interpreted as indirect evidence of voltage stability rather than as a direct measurement of the voltage across the gel.

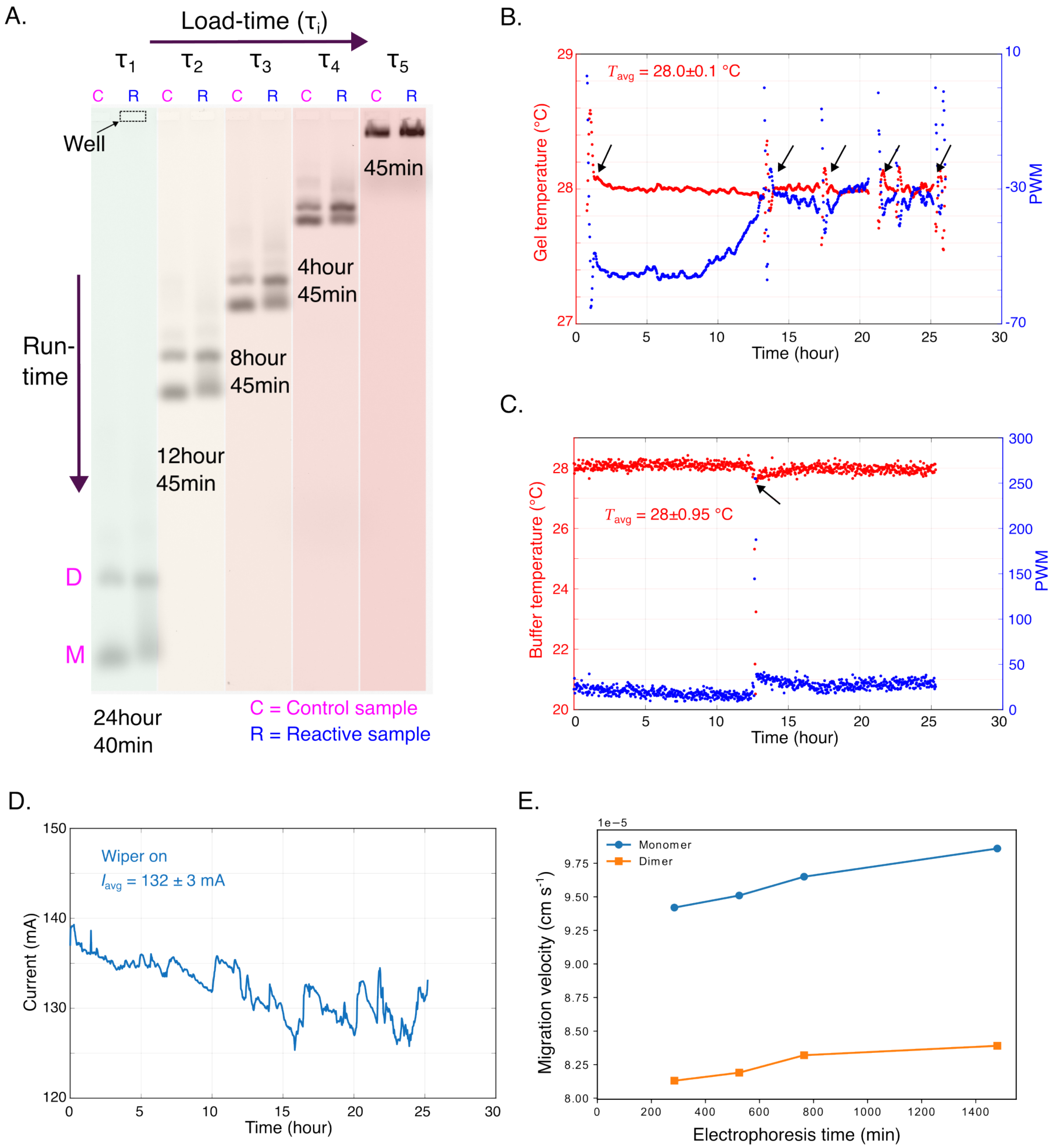

Figure S6: **Load-and-run gel electrophoresis at 28°C** (A) Gel electrophoresis of control and reactive DNA origami samples, performed at 50 V, 28°C and 20 mM $MgCl_2$ buffer. 'D' and 'M' represent dimer and monomer band respectively. (B) Gel temperature and the corresponding PWM data with time. The arrow signs indicate the transient response during sample loading in the gel. (C) Buffer temperature in the external reservoir and the corresponding PWM with time. The arrow sign indicates the transient response when the external buffer was replaced. (D) Electrophoresis current measured as a function of time during active wiping. (E) Migration velocities of monomer and dimer bands as a function of electrophoresis duration.

Table 1: Comparison of ARTGEL with representative electrophoresis platforms.

Platform	Primary purpose	Temperature control	Buffer circulation	Automated electrode cleaning	Long-duration operation	Open / customizable
**LabSmith uProcess + uEP01 [1]**	Conventional gel electrophoresis with programmable power and temperature regulation	Feedback-controlled through adjustment of electrophoresis power	Not an integral feature	Not an integral feature	Multi-hour operation demonstrated	Modular / programmable
**Helena SPIFE Touch [2]**	Rapid electrophoretic separation of temperature-sensitive samples	Integrated Peltier; chamber range 10–62 °C	Not reported / not required for primary application	No	Not reported; designed primarily for rapid separation	No; proprietary clinical platform
**ARTGEL**	Quantitative studies of temperature-sensitive reversible biomolecular association	Feedback-controlled thermoelectric regulation of gel temperature; demonstrated range 26–32 °C	Actively heated, continuously circulated external buffer reservoir	Yes	>24 h demonstrated	Yes
**Conventional electrophoresis platform**	Electrophoretic separation	Passive cooling / water-bath-assisted cooling	Supports circulation of buffer from an external reservoir	No	Not suitable for prolonged high-salt operation without measures to manage buffer and electrode conditions	Yes

9. Supplementary movie

Movie S1: **Operation of the ARTGEL instrument.** This movie demonstrates the operation of the ARTGEL instrument during electrophoresis (shown without insulation). Buffer circulation, active wiping, and real-time monitoring of temperature and current are shown in the video during device operation.

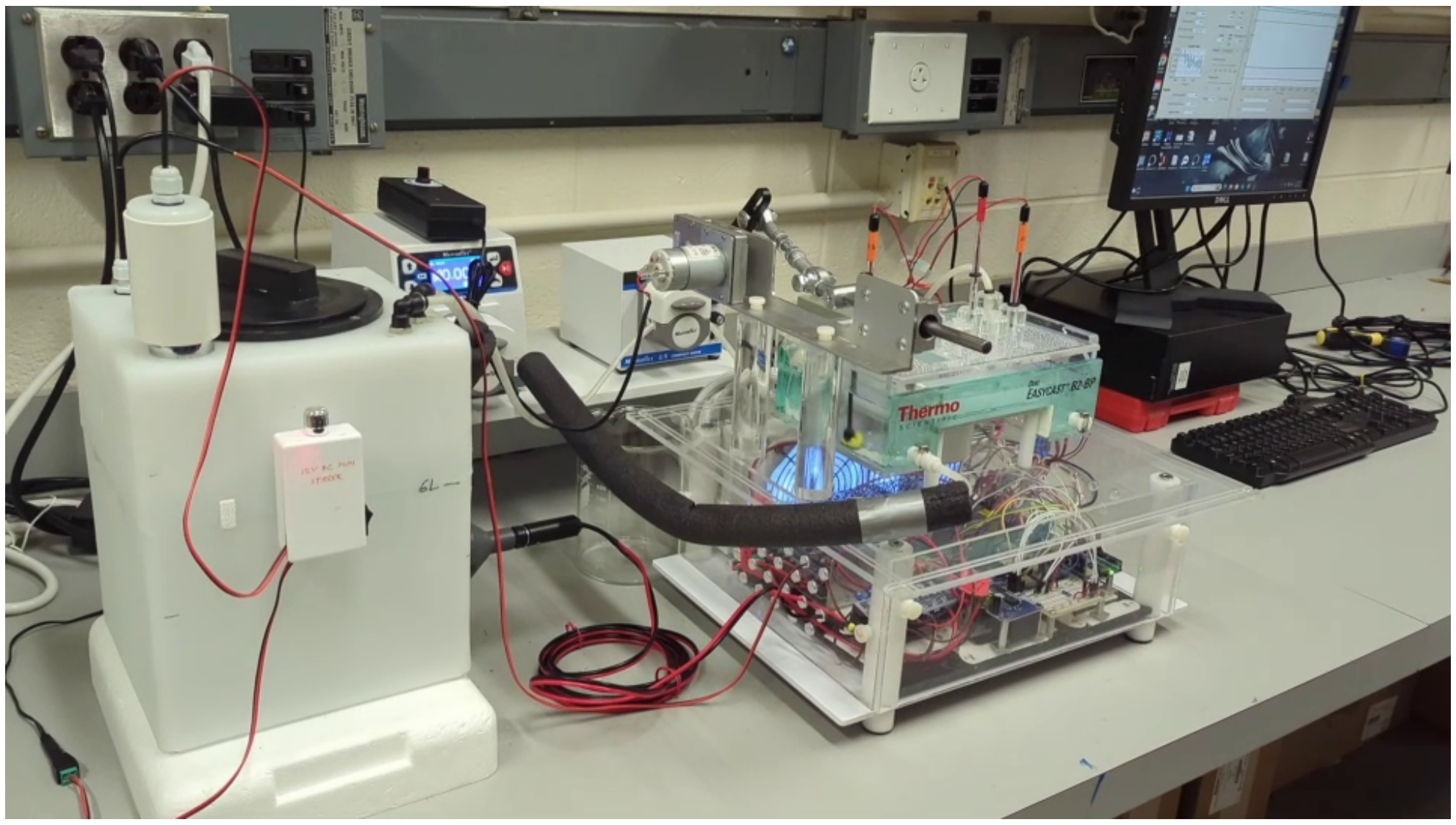

Figure S7: **Representative frame from Supplementary Movie S1 [thumbnail image].**

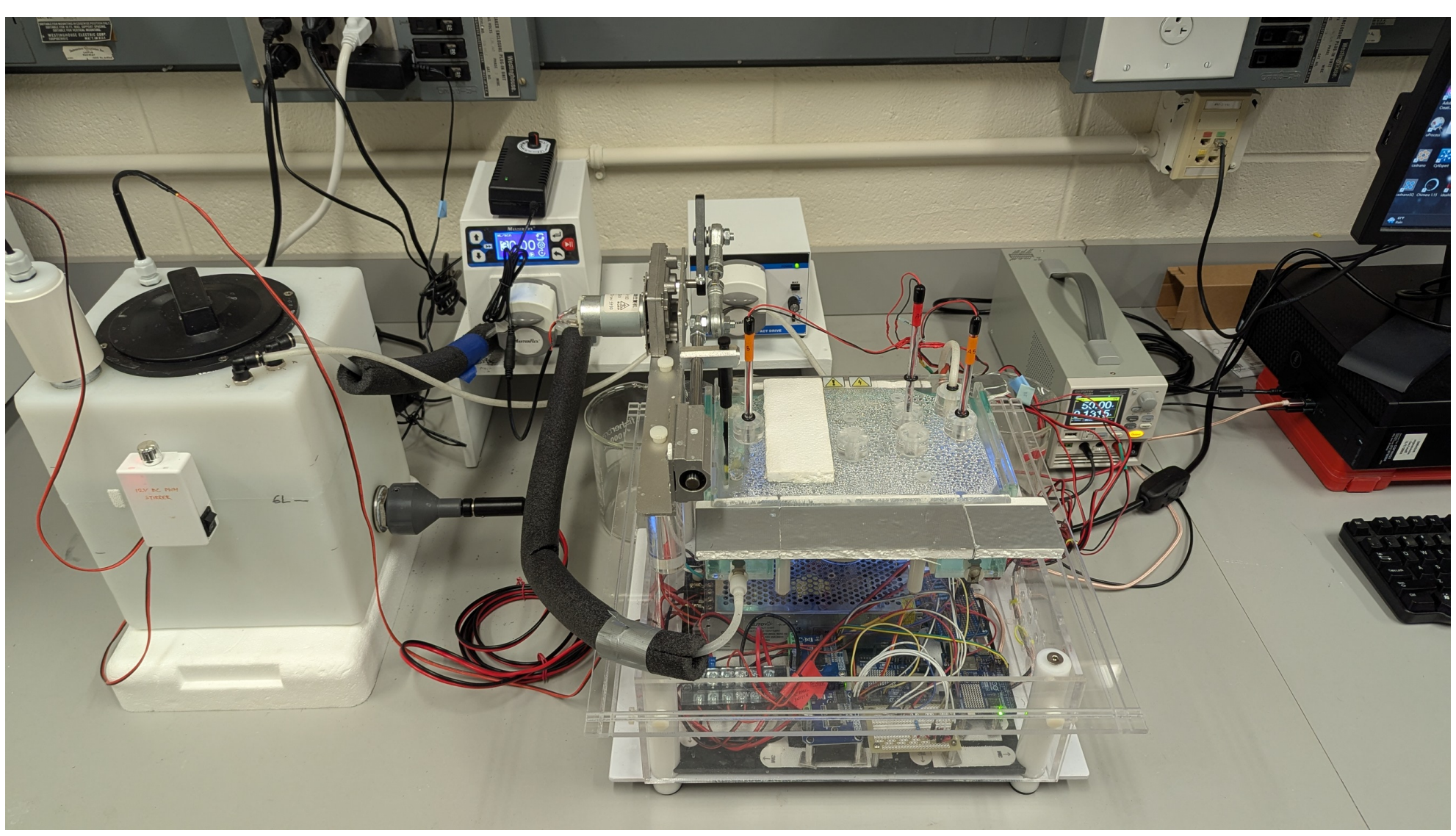

Figure S8: **ARTGEL instrument with insulated electrophoresis system.**

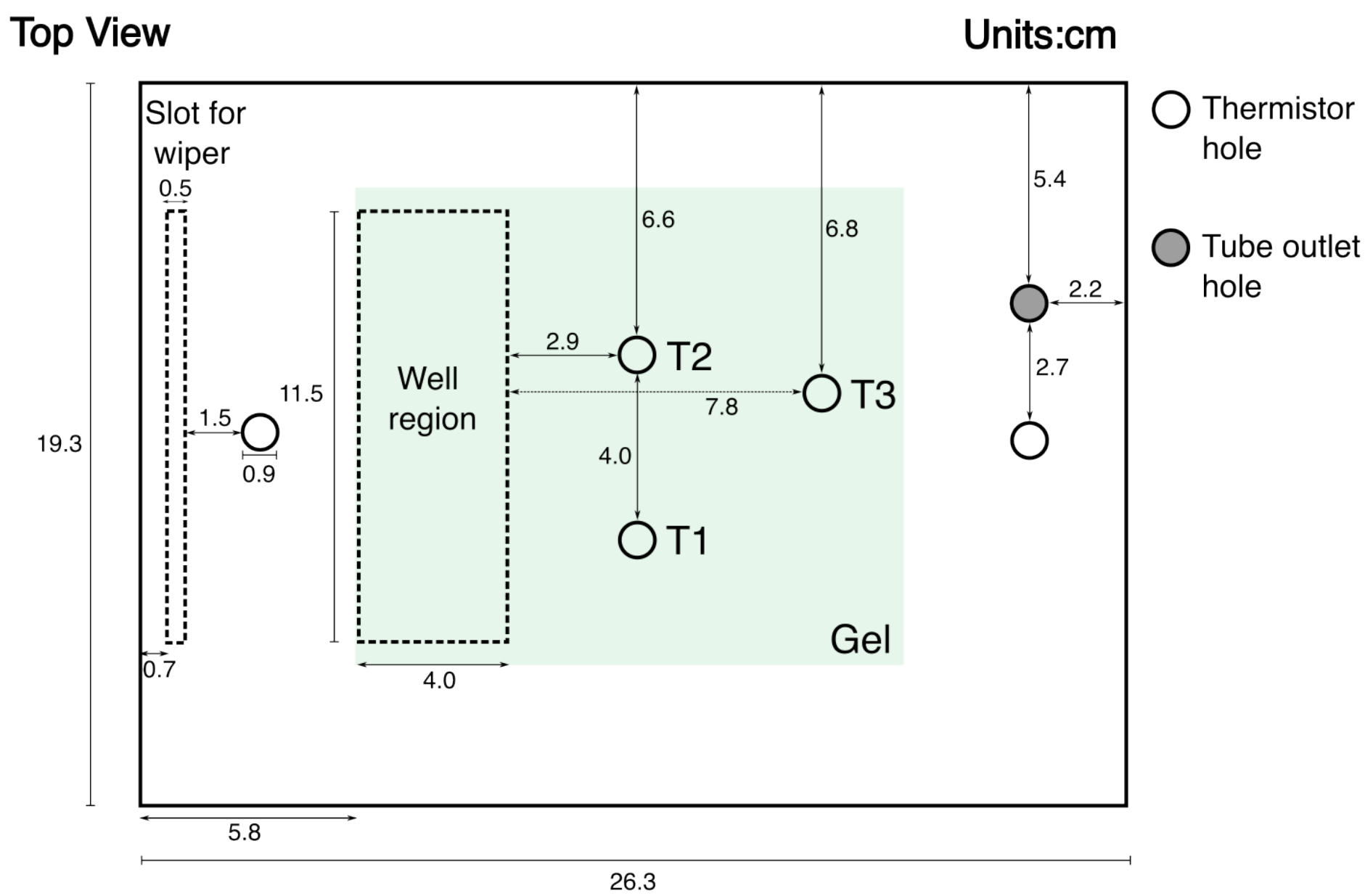

Figure S9: **Schematic of the top view of electrophoresis box lid.** Three Locations in the gel where temperature was measured simultaneously, labeled T1, T2, and T3, as shown in Fig. 10. Additional thermistor holes outside the gel footprint can be used to measure the buffer temperature. Tube outlet hole is used for buffer circulation.

References

[1] LabSmith. Gel electrophoresis temperature regulation with the uProcess uEP01 power supply. https://labsmith.com/labsmith-application/gel-electrophoresis-temperature-regulation-with-uprocess/, 2023.

[2] Helena Laboratories. Spife touch electrophoresis system. https://www.helena.com/spifetouch/.
